# Physical Activity and Cognitive Decline Among Older Adults: A Systematic Review and Meta-Analysis

**DOI:** 10.1001/jamanetworkopen.2023.54285

**Published:** 2024-02-01

**Authors:** Paula Iso-Markku, Sari Aaltonen, Urho M. Kujala, Hanna-Leena Halme, Daniel Phipps, Keegan Knittle, Eero Vuoksimaa, Katja Waller

**Affiliations:** 1 Institute for Molecular Medicine Finland, HiLIFE, University of Helsinki, Helsinki, Finland; 2 Helsinki University Hospital Diagnostic Center, Clinical Physiology and Nuclear Medicine, University of Helsinki and Helsinki University Hospital, Helsinki, Finland; 3 Faculty of Sport and Health Sciences, University of Jyväskylä, Jyväskylä, Finland

## Abstract

**Importance:**

Physical activity is associated with the risk for cognitive decline, but much of the evidence in this domain comes from studies with short follow-ups, which is prone to reverse causation bias.

**Objective:**

To examine how length of follow-up, baseline age, physical activity amount, and study quality modify the longitudinal associations of physical activity with cognition.

**Data Sources:**

Observational studies of adults with a prospective follow-up of at least 1 year, a valid baseline cognitive measure or midlife cohort, and an estimate of the association of baseline physical activity and follow-up cognition were sought from PsycInfo, Scopus, CINAHL, Web of Science, SPORTDiscus, and PubMed, with the final search conducted on November 2, 2022.

**Study Selection:**

Two independent researchers screened titles with abstracts and full-text reports.

**Data Extraction and Synthesis:**

Two reviewers independently assessed study quality and extracted data. Pooled estimates of association were calculated with random-effects meta-analyses. An extensive set of moderators, funnel plots, and scatter plots of physical activity amount were examined. This study is reported following the Preferred Reporting Items for Systematic Reviews and Meta-analyses (PRISMA) reporting guideline.

**Main Outcomes and Measures:**

Pooled estimates of the associations between physical activity and global cognition, as well as specific cognitive domains, were examined.

**Results:**

A total of 104 studies with 341 471 participants were assessed. Analysis of binary outcomes included 45 studies with 102 452 individuals, analysis of follow-up global cognition included 14 studies with 41 045 individuals, and analysis of change in global cognition included 25 studies with 67 463 individuals. Physical activity was associated with a decreased incidence of cognitive impairment or decline after correction for funnel plot asymmetry (pooled risk ratio, 0.97; 95% CI, 0.97-0.99), but there was no significant association in follow-ups longer than 10 years. Physical activity was associated with follow-up global cognition (standardized regression coefficient, 0.03; 95% CI, 0.02-0.03) and change in global cognition (standardized regression coefficient, 0.01; 95% CI, 0.01 to 0.02) from trim-and-fill analyses, with no clear dose-response or moderation by follow-up length, baseline age, study quality or adjustment for baseline cognition. The specific cognitive domains associated with physical activity were episodic memory (standardized regression coefficient, 0.03; 95% CI, 0.02-0.04) and verbal fluency (standardized regression coefficient, 0.05; 95% CI, 0.03-0.08).

**Conclusions and Relevance:**

In this meta-analysis of the association of physical activity with cognitive decline, physical activity was associated with better late-life cognition, but the association was weak. However, even a weak association is important from a population health perspective.

## Key Points

**Question:**

Is physical activity associated with cognitive decline?

**Findings:**

This systematic review and meta-analysis including a total of 104 studies with 341 471 participants found a weak association between baseline physical activity and follow-up global cognition that was evident also in episodic memory and verbal fluency domains. Neither study quality, follow-up length, baseline age, nor adjustment for preceding level of cognition moderated the association, and there was no clear dose-response association between the amount of physical activity and global cognition.

**Meaning:**

These findings suggest that physical activity might postpone cognitive decline at a population health level but only to a very small extent.

## Teaser

This systematic review and meta-analysis examines how length of follow-up, baseline age, physical activity amount, and study quality modify the longitudinal associations of physical activity with cognitionz.

## Introduction

Extensive research links physical activity with better cognitive outcomes across the lifespan^1^ and a decreased risk of dementia.^2,3^ Many different specific cognitive domains have been suggested to be associated with physical activity, but the evidence is inconsistent.^4,5^ Despite the optimism surrounding physical activity as a means to preserve or improve cognition, many recent high-quality interventional studies urge caution in claims linking cognitive benefits to physical activity,^6,7,8^ and most existing evidence comes from observational studies with short follow-ups and no information on preceding levels of cognition.

Observational prospective cohort studies can provide information on the association between risk factors and outcomes over very long follow-up periods, despite their inherent weaknesses. Earlier meta-analyses on physical activity and cognition have found a clear association between higher levels of physical activity and a decreased risk of subsequent cognitive decline^9,10,11,12^; however, these meta-analyses combined only studies with binary outcomes: cognitive impairment or decline or no cognitive impairment or decline^9,10,11,12^ and have examined only a few moderators. While binary outcomes are clinically relevant, modeling cognition as a continuous variable would improve statistical power.

This meta-analysis investigates whether physical activity is associated with global and domain-specific cognitive decline, examining cognition both categorically and continuously. Furthermore, we explore a possible dose-response association of physical activity with cognition and a broad range of possible moderators of this association. Taken together, this meta-analysis accounts for known limitations of research in this area to improve understanding of the association between physical activity participation and cognitive decline.

## Methods

This systematic review and meta-analysis was preregistered in PROSPERO (CRD42018083236) and follows the Preferred Reporting Items for Systematic Reviews and Meta-analyses (PRISMA) reporting guideline.^13^ The original registered plan was adapted according to data found. These changes are described in the eMethods in Supplement 1.

### Eligibility Criteria

#### Types of Studies and Participants

We included prospective cohort studies or case-control studies presenting an estimate of association between baseline physical activity and cognition after at least 1 year of follow-up. Participants were adults aged at least 20 years. We excluded cohorts with a specific disease, established dementia, or cognitive impairment at baseline. To be included, studies with baseline in later life (defined as mean or median age ≥55 years and maximum age ≥65 years or mean age within 1 SD of ≥60 years) were required to report a valid measure of cognition at baseline to reduce possible reverse causality.

#### Types of Exposure

Studies measuring contemporaneous physical activity with interviews, questionnaires, or devices were included. We excluded studies measuring retrospective physical activity level, cardiorespiratory or other fitness levels, single bouts of physical activity, physical activity extending over the follow-up, or statistical reallocations of physical activity.

#### Types of Outcomes

The association of baseline physical activity and cognition or specific cognitive domain (ie, executive function, episodic memory, processing speed, verbal fluency and naming, verbal ability, working memory, and visuospatial ability) at follow-up. We excluded studies reporting subjective estimates of cognition and studies in which cognition was based on registers of disability level.

#### Types of Reports

We included reports with full texts in English. We previously reported specifications to the exclusion and inclusion criteria in the screening and full-text review phases.^3^ Additional specifications in cases of disagreements were made during the update in 2022 to 2023. Changes are described in the eMethods in Supplement 1.

### Search Strategy

We conducted a systematic literature search in 6 databases (PubMed, PsycInfo, CINAHL, Scopus, SPORTDiscus, Web of Science). The last search was conducted November 2, 2022. Keywords included were *physical activity*, *sport*, *athletics*, *walking*, *physical training*, *cognition*, *cognitive*, *executive function*, *TELE* (telephone assessment for dementia), *TICS* (Telephone Interview for Cognitive Status), *MMSE* (Mini-Mental State Examination), *3-MS* (the Modified Mini-Mental State Examination), *memory*, *processing speed*, *verbal fluency*, *semantic fluency*, *reasoning*, *delayed recall*, *prospective*, *longitudinal*, *follow-up*, *observational*, and *cohort*. Individual additional articles known to the authors were also added. Example searches and further details of the search have been described in eTable 1 in Supplement 1).

### Study Selection

Two reviewers (P.I.-M. and S.A., D.P., or K.W.) independently assessed the eligibility of each study in 2 phases: title and abstract screening and full-text review. Disagreements were discussed, and a third reviewer (U.M.K.) made the final decision of inclusion if consensus was not reached. In cases of multiple studies reporting results for the same outcome from the same cohort from an overlapping time period, the study with a higher study quality, longer follow-up, larger sample, or which adjusted for baseline cognition was chosen.

### Quality Assessment

We developed a quality assessment tool with 3 tiered ranking (high, moderate, low) specifically for this meta-analysis.^3^ This tool provides high transparency of the quality assessment and accounts for the special characteristics of measuring physical activity and its association with an outcome with a long preclinical period extending over decades (cognitive impairment or dementia).^14^ One modification to this tool from our previous meta-analysis^3^ is described in the eMethods in Supplement 1. Two reviewers (P.I.-M. and D.P. or K.W.) conducted the quality review at the outcome level independently. Any disagreements were resolved with discussion.

### Data Extraction

Two reviewers (P.I.-M. and S.A., H.-L.H., D.P., or K.W.) extracted the following data: estimate of the association between physical activity and cognition or specific cognitive domain, measure of cognition, proportion of the sample who became cognitively impaired (or SD for continuous cognition outcomes), physical activity levels, sample size, country of origin, publication year, length of follow-up, age at baseline, work-related or leisure-time physical activity, confounders (cognition at baseline, chronic diseases, education, vascular risk factors, APOE ε4 allele), the number of confounders, device-based measure or self-report of physical activity, cohort, and follow-up rate. The extracted data were compared, and disagreements were solved by discussion. Follow-up rate was extracted by a single reviewer (P.I.-M.). Additional details of the data extraction are provided in eMethods and eTables 2-4 in Supplement 1 and eTables 1-12 in Supplement 2).

### Statistical Analysis

The results extracted from the original studies were risk ratios (RRs), hazard ratios, odds ratios, regression coefficients for cognition, number of participants with and without cognitive impairment at follow-up, regression coefficients for change in cognition, regression coefficients for rate of change in cognition, mean changes with 95% CIs, pre– and post–follow-up means and SDs, adjusted *P* values from analysis of covariance, means from repeated measures analysis of variance, and post–follow-up means and SDs with *P* values for a difference from a generalized linear regression model. We performed 3 separate sets of analyses for 3 different outcome types: a first set of analyses with risk of cognitive impairment or decline as the binary outcome, a second analysis with follow-up global cognition as a continuous outcome, and a third analysis with change in global cognition as a continuous outcome. Full details of transformations, standardizations, and the use of studies with cognition trajectories are described in eMethods in Supplement 1 and eTable 11 and eTable 12 in Supplement 2.

#### Binary Outcomes

We transformed hazard and odds ratios and number of participants with and without event at follow-up into risk ratios and pooled these risk ratios. In the main analyses, we compared all other physical activity levels with the lowest physical activity level.^3^

#### Continuous Outcomes

##### Follow-Up Cognition

We standardized unstandardized regression coefficients by dividing them by the SD of the outcome measure of cognition and multiplying them by the SD of physical activity.^15^ After standardization, we pooled the standardized regression coefficients for follow-up cognition with standardized mean differences in post–follow-up scores to yield us a pooled magnitude of association for physical activity and cognition. The basis for pooling standardized regression coefficients for cognition with standardized mean differences of the outcome post follow-up is that, although apparently different, the comparison of means of 2 independent groups measured at a single time with multiple regression analysis is mathematically equivalent with Cohen *d* from a *t* test for independent groups or between-participants analysis of variance with 2 groups.^15,16^ Thus, for 2 physical activity groups, the difference in cognition can be calculated with either of the 2 following equations: 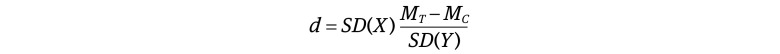


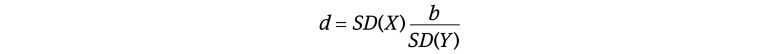
Where *d* indicates Cohen *d*; M_T_, the mean of the treatment group; M_C_, the mean of the control group; SD(Y), the pooled within-group SD of the outcome measure; SD(X), the SD of the independent variable; and *b*, the unstandardized regression coefficient for the association of the group.

##### Change in Cognition

In the set of analyses with change in cognition during the follow-up, we standardized the regression coefficients in a similar manner as for follow-up global cognition, but instead used the SD of cognition at baseline (between-participant variability at baseline).^16,17^ We pooled all standardized regression coefficients for change with standardized mean differences of change^16^ to yield 1 estimate for the association of physical activity with change in cognition.

#### Pooling the Studies

We used random-effects models with inverse variance as the weighting method for our meta-analysis. Heterogeneity was estimated with DerSimonian and Laird method. The possibility of publication bias was estimated with contour-enhanced funnel plots. We used the trim-and-fill method with run estimator to correct any funnel plot asymmetry. We present the results as pooled RRs with 95% CIs for studies with binary outcomes and as pooled standardized regression coefficients with 95% CIs for studies with continuous outcomes.

The main analyses were risk of cognitive impairment or decline (ie, analysis of binary outcomes), standardized regression coefficient for follow-up global cognition, and standardized regression coefficient for change in global cognition during the follow-up. In addition, we performed separate analyses for the following specific cognitive domains: executive function, working memory, processing speed, episodic memory, verbal fluency and naming, verbal ability, and visuospatial ability. In the sensitivity analyses, we examined categorical moderators with subgroup analyses and continuous moderators with meta-regressions. The following moderators were examined: follow-up length, baseline and follow-up age, study quality, type of physical activity, measurement of physical activity, validity of physical activity measurement, sample size, number of confounders, measurement of cognition (at least 1 neuropsychological test vs dementia screening tool), follow-up rate, and adjustment for preceding level of cognition, education, chronic diseases, APOE ε4 allele, and other vascular risk factors.

In addition, we examined a possible dose-response association between physical activity and global cognition with scatter plots and fitted lines. The details of this analysis are described in the eMethods in Supplement 1. *P* values were 2-sided, and statistical significance was set at *P* = .05. All analyses were performed with Stata statistical software version 18.0 (StataCorp). Data were analyzed from January to August 2023, with a final analysis in December 2023.

## Results

**Figure 1.**
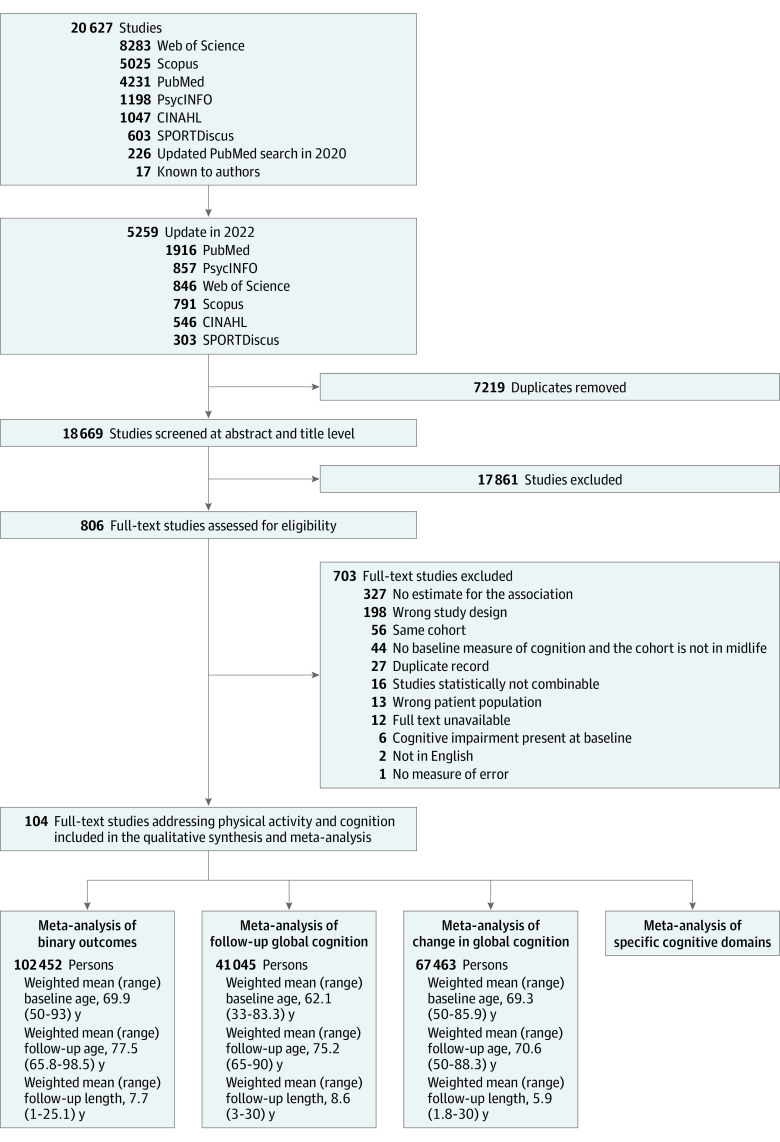
Study Selection Flowchart

The searches yielded 18 669 articles, of which 17 861 were excluded in title and abstract review phase and 703 were excluded in full-text review (Figure 1). This resulted in 104 included studies addressing physical activity and cognition, assessing a total of 341 471 participants. Of these, 45 had a binary outcome,^18,19,20,21,22,23,24,25,26,27,28,29,30,31,32,33,34,35,36,37,38,39,40,41,42,43,44,45,46,47,48,49,50,51,52,53,54,55,56,57,58,59,60,61,62^ 14 addressed follow-up global cognition,^63,64,65,66,67,68,69,70,71,72,73,74,75,76^ 25 addressed change or rate of change in global cognition,^74,76,77,78,79,80,81,82,83,84,85,86,87,88,89,90,91,92,93,94,95,96,97,98,99^ and 37 addressed physical activity and a specific cognitive domain of cognition.^20,25,47,57,64,66,71,74,75,78,85,89,91,92,95,98,99,100,101,102,103,104,105,106,107,108,109,110,111,112,113,114,115,116,117,118,119,120,121,122^ The quality assessments of all included studies are presented in eFigure 1 in Supplement 1.

### Physical Activity and Cognition With Binary Outcomes

Among 45 studies^18,19,20,21,22,23,24,25,26,27,28,29,30,31,32,33,34,35,36,37,38,39,40,41,42,43,44,45,46,47,48,49,50,51,52,53,54,55,56,57,58,59,60,61,62^ with a binary outcome, 1 study^29^ was of high quality, 13 of moderate quality,^18,22,24,28,32,37,38,41,43,46,47,55,59^ and 31 of low quality.^19,20,21,23,25,26,27,30,31,33,34,35,36,39,40,42,44,45,48,49,50,51,52,53,54,56,57,58,60,61,62^ Physical activity was associated with a decreased risk of cognitive impairment or decline (RR, 0.89; 95% CI, 0.86-0.92; *I*^2^ = 69.6%) (Table 1 and Table 2; eFigure 2 in Supplement 1). Contour-enhanced funnel plot showed asymmetry (eFigure 3 in Supplement 1) due to reasons other than publication bias, like study quality. Trim-and-fill analysis correcting for this asymmetry showed a weaker association between physical activity and risk of cognitive impairment (RR, 0.97; 95% CI, 0.97-0.99) (Table 1). From the extensive set of moderators, only quality of physical activity measurement, sample size, follow-up rate, and length of follow-up significantly moderated the association between physical activity and cognition (Table 1; eTable 4 and eFigures 4-6 in Supplement 1). There was no significant association in follow-ups longer than 10 years (Table 1), while higher quality physical activity measurements and higher follow-up rate were associated with better cognition (eTable 4 in Supplement 1). The association was weaker in larger studies, but this result seemed largely driven by a few studies with very large sample sizes (>10 000 persons) (eTable 4 and eFigure 4 in Supplement 1).

**Table 1.** Main Analysis of the Association of Physical Activity With Cognition

Analysis	Estimate (95% CI)	*I*^2^, %	No. of studies combined	Regression coefficient of the meta-regression	*P* value
**Physical activity: binary outcomes**						
Overall	0.89 (0.86 to 0.92)^a^	69.6	45	NA	NA
Meta trim-and-fill analysis	0.97 (0.97 to 0.99)^a^	NA	NA	NA	NA
Baseline age (continuous)	NA	NA	NA	0.0002	.93
Follow-up length					
Continuous	NA	NA	NA	0.008	.007
Categorical, y					
<5	0.81 (0.76 to 0.86)^a^	74.0	22	NA	NA
5-9	0.90 (0.86 to 0.95)^a^	58.7	13	NA	NA
10-14	1.06 (0.95 to 1.17)^a^	16.2	4	NA	NA
≥15	0.93 (0.82 to 1.05)^a^	77.2	6	NA	NA
Study quality (continuous)^b^	NA	NA	NA	0.03	.41
Adjusting for preceding level of cognition	NA	NA	NA	NA	NA
No	0.89 (0.85 to 0.93)^a^	69.2	31	NA	.23
Yes	0.85 (0.79 to 0.91)^a^	71.6	14	NA	
**Physical activity and follow-up global cognition**						
Overall	0.025 (0.004 to 0.047)^c^	75.8	14	NA	NA
Meta trim- and fill analysis	0.025 (0.017 to 0.034)^c^	NA	NA	NA	NA
Age at baseline (continuous)	NA	NA	NA	−0.0006	.94
Follow-up length (continuous)	NA	NA	NA	0.0008	.69
Study quality (continuous)^b^	NA	NA	NA	−0.03	.35
Adjustment for preceding level of cognition	NA	NA	NA	NA	NA
No	0.005 (−0.033 to 0.043)^c^	76.6	8	NA	.14
Yes	0.041 (0.012 to 0.071)^c^	77.2	6	NA	
**Physical activity and change in global cognition**						
Overall	0.016 (0.002 to 0.030)^c^	67.4	25	NA	NA
Meta trim- and fill analysis	0.013 (0.008 to 0.018)^c^	NA	NA	NA	NA
Age at baseline (continuous)	NA	NA	NA	−0.0005	.75
Follow-up length (continuous)	NA	NA	NA	−0.0009	.69
Study quality (continuous)^b^	NA	NA	NA	0.003	.90
Adjustment for preceding level of cognition	NA	NA	NA	NA	
No	0.023 (−0.010 to 0.057)^c^	79.0	14	NA	.64
Yes	0.015 (0.010 to 0.020)^c^	0	11	NA	

Abbreviation: NA, not applicable.

^a^ Expressed as pooled risk ratios.

^b^ Study quality was assessed with a quality assessment tool we developed (range, 1-3; higher score denotes worse quality). Further details are provided in the eMethods in Supplement 1.

^c^ Expressed as pooled standardized regression coefficients.

**Table 2.** Studies Assessing Physical Activity and Cognition as Binary Outcomes

Study	Risk ratio 95% (CI)	Weight, %
Beauchet et al,^52^ 2020	1.21 (0.65-2.19)	0.29
Brunner et al,^18^ 2017		
Moderately active	0.99 (0.89-1.11)	3.14
Sufficiently active	0.89 (0.79-1.01)	2.88
Chen et al,^19^ 2016	1.08 (0.93-1.27)	2.33
Clark et al,^20^ 2016		
Second quintile	0.83 (0.55-1.24)	0.60
Third quintile	1.27 (0.90-1.77)	0.81
Fourth quintile	0.95 (0.65-1.35)	0.72
Fifth quintile	0.77 (0.50-1.15)	0.58
de Frias et al,^21^ 2014	1.08 (0.93-1.26)	2.42
de Looze et al,^51^ 2022	0.93 (0.85-1.02)	3.50
Dupré et al,^55^ 2020		
Second category	1.01 (0.81-1.29)	1.43
Third category	0.75 (0.50-1.15)	0.57
Elwood et al,^22^ 2013	0.79 (0.64-0.96)	1.73
Etgen et al,^23^ 2010		
High activity	0.54 (0.32-0.87)	0.42
Moderate activity	0.59 (0.36-0.95)	0.44
Fassier et al,^57^ 2022		
Tertile 2	0.94 (0.75-1.19)	1.42
Tertile 3	0.81 (0.63-1.02)	1.37
Gao et al,^24^ 2017	1.19 (1.07-1.31)	3.26
He et al,^59^ 2021	0.78 (0.64-0.94)	1.89
Hildreth et al,^25^ 2014	0.75 (0.55-1.00)	1.00
Ho et al,^26^ 2001	0.69 (0.55-0.88)	1.42
Hughes et al,^27^ 2015	0.42 (0.25-0.72)	0.37
Infurna et al,^28^ 2016	1.06 (1.02-1.11)	4.28
Iso-Markku et al,^29^ 2016	0.78 (0.66-0.94)	2.00
Iwasa et al,^30^ 2012	0.97 (0.76-1.24)	1.31
Kim et al,^31^ 2011	0.81 (0.70-0.95)	2.38
Krell-Roesch et al,^62^ 2021	0.86 (0.74-1.00)	2.41
Laurin et al,^32^ 2001		
Men with high activity	0.70 (0.41-1.18)	0.37
Men with low activity	0.67 (0.32-1.33)	0.21
Men with moderate activity	0.85 (0.55-1.30)	0.54
Women with high activity	0.49 (0.27-0.91)	0.29
Women with low activity	0.71 (0.43-1.14)	0.44
Women with moderate activity	0.57 (0.38-0.83)	0.64
Lee et al,^33^ 2013		
Quartile 2	0.76 (0.53-1.10)	0.72
Quartile 3	0.73 (0.50-1.06)	0.69
Quartile 4	0.62 (0.42-0.91)	0.66
Leung et al,^34^ 2011	0.97 (0.95-0.99)	4.52
Lipnicki et al,^35^ 2017	0.96 (0.89-1.04)	3.66
Lytle et al,^36^ 2004		
High exercise	0.62 (0.44-0.88)	0.76
Low exercise	0.83 (0.66-1.05)	1.42
McGarrigle et al,^60^ 2022	1.00 (0.98-1.02)	4.55
Middleton et al,^37^ 2011		
Highest tertile of device-measured activity	0.10 (0.01-0.80)	0.02
Lowest tertile of device-measured activity	0.30 (0.07-1.21)	0.05
Min et al,^56^ 2018	0.67 (0.47-0.96)	0.73
Newman et al,^38^ 2009		
>1890 kcal	1.42 (0.56-3.18)	0.14
1-270 kcal	1.07 (0.39-2.63)	0.12
271-810 kcal	1.28 (0.49-2.93)	0.14
811-890 kcal	0.70 (0.26-1.80)	0.12
Niti et al,^39^ 2008	0.88 (0.78-1.01)	2.69
Pignatti et al,^40^ 2002	0.27 (0.09-0.83)	0.09
Pitrou et al,^61^ 2022	0.94 (0.59-1.51)	0.46
Ramoo et al,^53^ 2022		
Quartile 2	0.69 (0.47-1.02)	0.65
Quartile 3	0.99 (0.68-1.50)	0.63
Quartile 4	0.53 (0.38-0.76)	0.78
Shih et al,^41^ 2017	0.71 (0.50-0.99)	0.81
Stewart et al,^42^ 2003	0.84 (0.58-1.19)	0.74
Strozza et al,^54^ 2020		
Light physical activity	0.87 (0.61-1.19)	0.84
Heavy physical activity	0.69 (0.42-1.11)	0.44
Sumic et al,^43^ 2007		
Men	0.94 (0.34-1.64)	0.17
Women	0.17 (0.04-0.52)	0.07
Thompson et al,^58^ 2022	1.37 (0.70-2.71)	0.23
Verdelho et al,^44^ 2012	0.72 (0.57-0.92)	1.36
Verghese et al,^48^ 2006	0.97 (0.94-1.01)	4.37
Verghese et al,^49^ 2009	0.99 (0.96-1.03)	4.44
Wang et al,^50^ 2006	0.96 (0.84-1.09)	2.68
Woodard et al,^45^ 2012	0.99 (0.38-2.55)	0.12
Yaffe et al,^46^ 2001		
Highest quartile	0.86 (0.78-0.95)	3.26
Second quartile	0.95 (0.86-1.04)	3.35
Third quartile	0.88 (0.80-0.98)	3.26
Zhu et al,^47^ 2017		
Quartile 2	0.65 (0.49-0.85)	1.16
Quartile 3	0.54 (0.39-0.75)	0.86
Quartile 4	0.58 (0.40-0.82)	0.74
Overall (*I*^2^ = 69.6%; *P* < .001)	0.89 (0.86-0.92)	100

**Figure 2.**
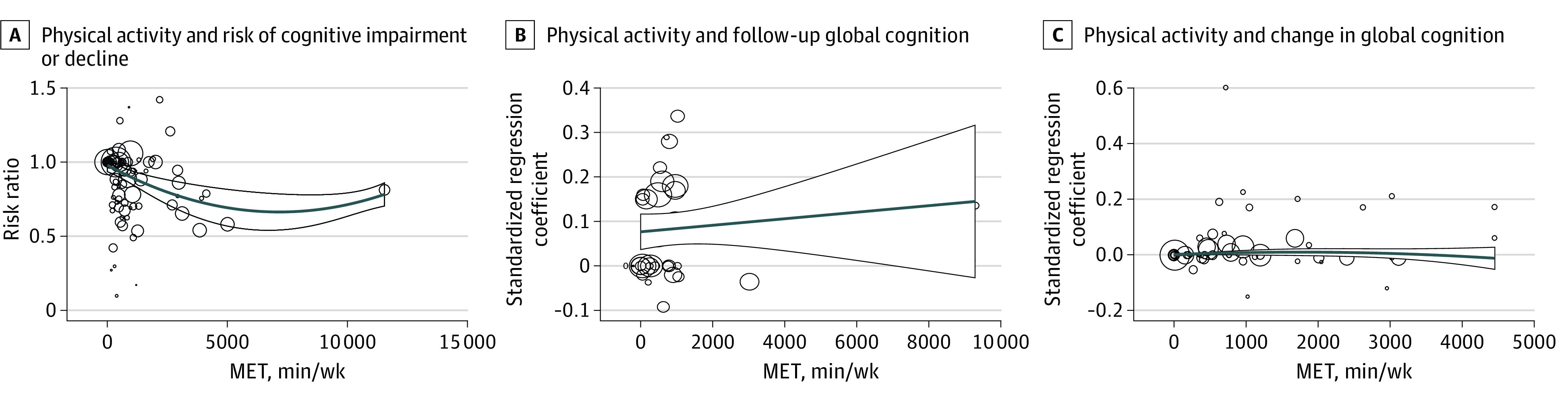
Assessment of Dose-Response Association of Physical Activity With Cognition MET indicates metabolic equivalent task; circles, individual studies; size of circles, weight of studies; blue line, estimate; outline, 95% CI.

Contour-enhanced funnel plot showed asymmetry (eFigure 3 in Supplement 1) due to reasons other than publication bias, like study quality. Trim-and-fill analysis correcting for this asymmetry showed a weaker association between physical activity and risk of cognitive impairment (RR, 0.97; 95% CI, 0.97-0.99) (Table 1). The amount of physical activity had a larger inverse association with cognitive impairment or decline until 5000 metabolic equivalent of task–minutes per week (ie, 16 hours of moderate to vigorous physical activity per week) (Figure 2A).

### Physical Activity and Follow-Up Global Cognition

**Figure 3.**
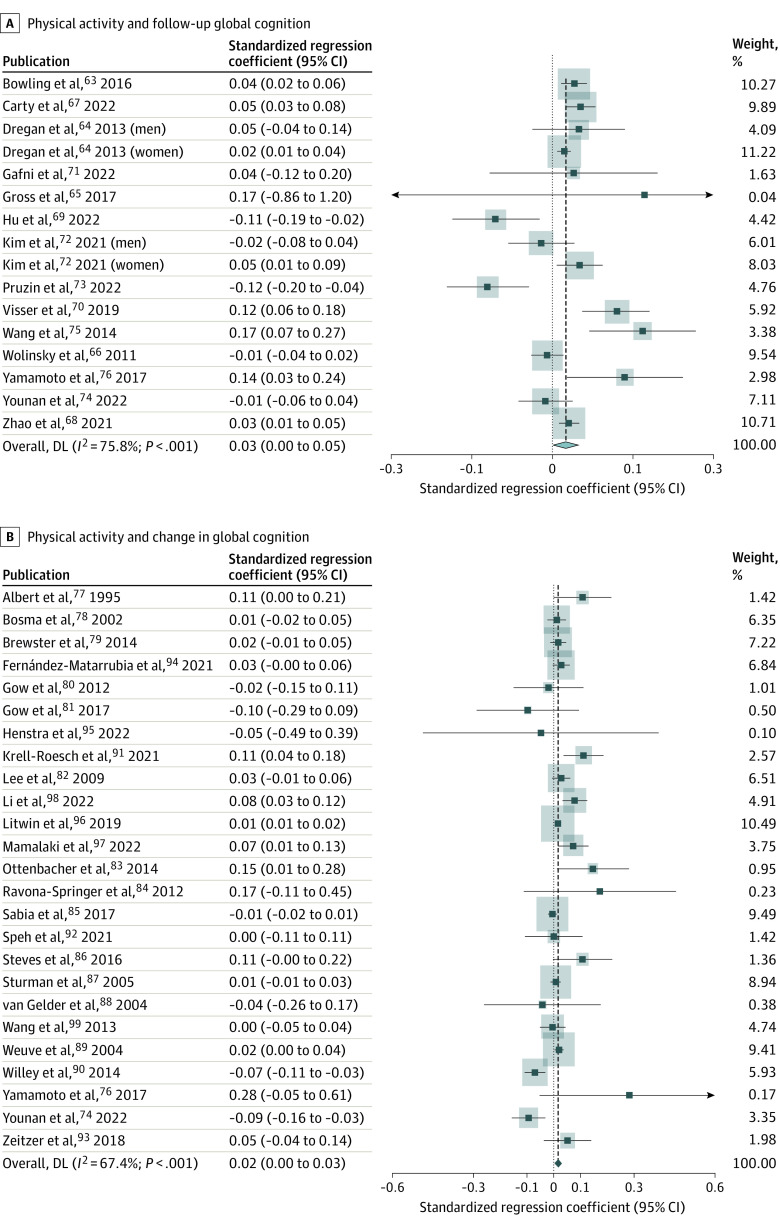
Assessment of Association of Physical Activity With Cognition Using Continuous Outcomes Shaded areas indicate study weight.

Among 14 studies that assessed follow-up global cognition, there were no high-quality studies, 4 moderate-quality studies,^63,65,67,71^ and 10 low-quality studies.^64,66,68,69,70,72,73,74,75,76^ Pooled analysis showed a significant positive association between physical activity and follow-up global cognition (pooled β = 0.025; 95% CI, 0.004-0.047) (Table 1 and Figure 3A). The heterogeneity between the results from different studies was large (*I*^2^ = 75.8%) (Table 1). None of the examined moderators significantly moderated the association between physical activity and cognition (Table 1; eTable 5 in Supplement 1). The funnel plot showed minimal asymmetry (eFigure 7 in Supplement 1), and the trim-and-fill analysis correcting for this asymmetry showed a very similar pooled standardized regression coefficient for physical activity and follow-up global cognition with narrower CIs (β = 0.025; 95% CI, 0.017-0.034) (Table 1). No dose-response association was seen (Figure 2B).

### Physical Activity and Change in Global Cognition

Among 25 studies that assessed change in global cognition, there were no high-quality studies, 5 moderate-quality studies,^83,87,88,89,99^ and 20 low-quality studies.^74,76,77,78,79,80,81,82,84,85,86,90,91,92,93,94,95,96,97,98^ Pooled analysis of all studies addressing physical activity and change in global cognition showed a significant positive association (pooled β = 0.016; 95% CI, 0.002-0.030) (Table 1 and Figure 3B). The heterogeneity among the results from different studies was large (I^2^ = 67.4%) (Table 1). The funnel plot showed at most minimal asymmetry (eFigure 8 in Supplement 1). Trim-and-fill analysis showed a similar pooled standardized regression coefficient as the main analysis with a narrower CI (β = 0.013; 95% CI, 0.008-0.018) (Table 1). None of the moderators examined significantly moderated the association between physical activity and change in cognition (Table 1; eTable 6 in Supplement 1). There was no dose-response association (Figure 2C).

### Physical Activity and Specific Cognitive Domains

There were no significant associations between physical activity and any of the specific cognitive domains in studies with binary outcomes (eTable 7 in Supplement 1). In the studies of physical activity and follow-up cognition, physical activity was associated with minimally better executive function (standardized regression coefficient, 0.05, 95% CI 0.01 to 0.09), episodic memory (standardized regression coefficient, 0.03; 95% CI, 0.02-0.04), and verbal fluency (standardized regression coefficient, 0.05; 95% CI, 0.03-0.08). There was only 1 study on physical activity and verbal ability.^117^ In the studies of physical activity and change in cognition, physical activity was significantly and minimally associated with episodic memory and verbal fluency. Adjustment for the preceding level of cognition did not significantly modify the associations between physical activity and specific cognitive domains. The only exception was verbal fluency, for which the association was significant only when adjusting for preceding level of cognition in studies of change in cognition and significant, in binary studies, only when not adjusting for preceding level of cognition (eTable 8 in Supplement 1).

## Discussion

This systematic review and meta-analysis found only minimal associations between physical activity and cognition. These very small estimates are more in line with a recent umbrella review of randomized clinical trials showing very small effects sizes between physical activity and cognition^6^ than with earlier meta-analyses of observational studies on physical activity and cognition, which showed moderate associations.^9,10,11,12^ For comparison, a 2018 study found that each 1-year increase in age was associated with a 0.037-SD decrease in global cognition.^123^ The identified weak association between physical activity and cognition was persistent, regardless of the preceding level of cognition or cohort age, which is in line with our previous meta-analysis of physical activity and dementia.^3^ Although the pooled standardized magnitudes of association were very small,^15,124^ they are significant in a population health perspective for the potential to postpone the multifactorial diseases causing dementia.

Our results indicate a dose-response association between physical activity and cognition among studies with binary cognition outcomes. This association was moderated by follow-up length, follow-up rate, physical activity measurement type, and physical activity measurement quality, but funnel plots detected possible bias in this set of studies. On the contrary, while not revealing possible bias, our meta-analysis of studies with continuous outcomes found neither a dose-response association nor any significant moderation. This contradicts our earlier meta-analysis of physical activity and dementia that found a dose-response association^3^ and also other meta-analyses of physical activity and dementia.^125,126^

Beyond this, study quality did not significantly moderate any of the associations. For a study to be rated as high quality in our assessment, it needed to include a follow-up of more than 10 years,^85^ a measurement of baseline cognition, and very high participation and follow-up rates—all factors that are necessary to accurately examine longitudinal associations between physical activity and cognition. Further high-quality research is needed.

The analysis of physical activity and specific cognitive domains revealed similar weak associations for episodic memory and verbal fluency as the main analysis (pooled standardized regression coefficients between 0.02 and 0.05). The results for executive function were mixed between analyses of follow-up and change. The CIs for the associations were wider for verbal ability, working memory, processing speed, and visuospatial ability, but the data for these analyses were scarcer and explain at least partly the wider CIs.

Our meta-analysis has many strengths. To our knowledge, no other meta-analysis of observational studies has examined continuous outcomes or specific cognitive domains before. In addition, we combined the data of 6 times more individuals (>300 000) than any previous meta-analysis on the topic^9,10,11,12^ and examined more possible moderators.

### Limitations

This meta-analysis has limitations. High-quality studies were rare and data examining midlife physical activity and midlife cognition were scarce. Thus, our meta-analysis mainly provides evidence on how physical activity was associated with cognitive aging, not cognition in midlife. In retrospect, excluding studies without valid measures of cognition at baseline perhaps limited power to detect adjustment for baseline cognition as a moderator. We also did not assess whether studies accounted for practice effects when measuring cognition. Low study quality and imprecise study-level measures of physical activity and cognition limit the robustness of our dose-response analyses.

## Conclusions

This systematic review and meta-analysis found that the association between physical activity and cognitive decline was very small, with no evident dose-response association. With that said, even weak associations can be clinically significant from a population health perspective when physical activity continues over decades. It should also be noted that very few high-quality studies were included. Further high-quality cohort studies with follow-ups longer than 10 to 20 years, fine-grained measures of physical activity and cognition at baseline, and high participation and follow-up rates are needed to solidify the evidence base in this area.

## Notes

Supplement 1.
eMethods.
**eTable 1.** Search Strategy **eTable 2.** Definitions of Cognitive Impairment and Decline **eTable 3.** Tests Used to Assess Continuous Global Cognition, Verbal Fluency and Naming, Working Memory, Verbal Ability and Visuo-spatial Ability **eTable 4.** Tests Used to Assess Episodic Memory, Executive Function and Processing Speed **eFigure 1.** Quality of the Studies **eTable 5.** Physical Activity and Cognition, Binary Outcomes, Supplementary Analyses **eFigure 2.** Physical Activity and Cognitive Impairment or Decline, Forest Plot **eFigure 3.** Physical Activity and Cognition, Binary Outcome, Contour-Enhanced Funnel Plot **eFigure 4.** Physical Activity and Cognition, Binary Outcomes by Sample Size **eFigure 5.** Physical Activity and Cognition, Binary Outcomes by Length of the Follow-Up **eFigure 6.** Physical Activity and Cognition, Binary Outcomes by Follow-Up Rate (Studies Not Reporting Follow-Up Rate Are Excluded) **eTable 6.** Physical Activity and Follow-Up Cognition: Supplementary Analyses **eFigure 7.** PA and Follow-Up Cognition, Contour-Enhanced Funnel Plot **eTable 7.** PA and Change in Cognition: Supplementary Analyses **eFigure 8.** PA and Change in Cognition, Contour-Enhanced Funnel Plot **eTable 8.** PA and Specific Cognitive Domains in Studies Adjusting for Preceding Level of Cognition
eReferences.

Supplement 2. **eTable 1.** Quality Review **eTable 2.** Binary Outcomes **eTable 3.** Continuous Outcomes **eTable 4.** Executive Function **eTable 5.** Episodic Memory **eTable 6.** Processing Speed **eTable 7.** Verbal Fluency and Naming **eTable 8.** Working Memory **eTable 9.** Verbal Ability **eTable 10.** Visuospatial Ability **eTable 11.** Dose Analysis: Binary Outcomes **eTable 12.** Dose Analysis: Continuous Outcomes

Supplement 3.
Data Sharing Statement
