# Supplementary material for: Physical Activity and Cognitive Decline Among Older Adults: A Systematic Review and Meta-Analysis
Source: JAMA Netw Open. 2024 Feb 1;7(2):e2354285. doi: 10.1001/jamanetworkopen.2023.54285 (PMC10835510; doi:10.1001/jamanetworkopen.2023.54285)
Supplement: Supplement 1. — eMethods. eTable 1. Search Strategy eTable 2. Definitions of Cognitive Impairment and Decline eTable 3. Tests Used to Assess Continuous Global Cognition, Verbal Fluency and Naming, Working Memory, Verbal Ability and Visuo-spatial Ability eTable 4. Tests Used to Assess Episodic Memory, Executive Function and Processing Speed eFigure 1. Quality of the Studies eTable 5. Physical Activity and Cognition, Binary Outcomes, Supplementary Analyses eFigure 2. Physical Activity and Cognitive Impairment or Decline, Forest Plot eFigure 3. Physical Activity and Cognition, Binary Outcome, Contour-Enhanced Funnel Plot eFigure 4. Physical Activity and Cognition, Binary Outcomes by Sample Size eFigure 5. Physical Activity and Cognition, Binary Outcomes by Length of the Follow-Up eFigure 6. Physical Activity and Cognition, Binary Outcomes by Follow-Up Rate (Studies Not Reporting Follow-Up Rate Are Excluded) eTable 6. Physical Activity and Follow-Up Cognition: Supplementary Analyses eFigure 7. PA and Follow-Up Cognition, Contour-Enhanced Funnel Plot eTable 7. PA and Change in Cognition: Supplementary Analyses eFigure 8. PA and Change in Cognition, Contour-Enhanced Funnel Plot eTable 8. PA and Specific Cognitive Domains in Studies Adjusting for Preceding Level of Cognition eReferences. [file jamanetwopen-e2354285-s001.pdf]

Supplemental Online Content

Iso-Markku P, Aaltonen S, Kujala UM, et al. Physical activity and cognitive decline among older adults. *JAMA Netw Open*. 2024;7(2):e2354285. doi:10.1001/jamanetworkopen.2023.54285

eMethods.

eTable 1. Search Strategy

eTable 2. Definitions of Cognitive Impairment and Decline

eTable 3. Tests Used to Assess Continuous Global Cognition, Verbal Fluency and Naming, Working Memory, Verbal Ability and Visuo-spatial Ability

eTable 4. Tests Used to Assess Episodic Memory, Executive Function and Processing Speed

eFigure 1. Quality of the Studies

eTable 5. Physical Activity and Cognition, Binary Outcomes, Supplementary Analyses

eFigure 2. Physical Activity and Cognitive Impairment or Decline, Forest Plot

eFigure 3. Physical Activity and Cognition, Binary Outcome, Contour-Enhanced Funnel Plot

eFigure 4. Physical Activity and Cognition, Binary Outcomes by Sample Size

eFigure 5. Physical Activity and Cognition, Binary Outcomes by Length of the Follow-Up

eFigure 6. Physical Activity and Cognition, Binary Outcomes by Follow-Up Rate (Studies Not Reporting Follow-Up Rate Are Excluded)

eTable 6. Physical Activity and Follow-Up Cognition: Supplementary Analyses

eFigure 7. PA and Follow-Up Cognition, Contour-Enhanced Funnel Plot

eTable 7. PA and Change in Cognition: Supplementary Analyses

eFigure 8. PA and Change in Cognition, Contour-Enhanced Funnel Plot

eTable 8. PA and Specific Cognitive Domains in Studies Adjusting for Preceding Level of Cognition

eReferences.

This supplemental material has been provided by the authors to give readers additional information about their work.

© 2023 Iso-Markku P et al. *JAMA Network Open.*

1. eMethods

This meta-analysis examines the relationship between PA and cognition. A separate pre-registered meta-analysis examined the relationship between PA and the incidence of all-cause dementia, Alzheimer's disease, and vascular dementia. The study plan originally encompassed both meta-analyses and was submitted to PROSPERO 2.12.2017 (prior to the first search of the review 11.12.2017). The registration was accepted 8.1.2018. The plan of the search has been described in PROSPERO (registration number CRD42018083236) and addressed the relationship of PA with both cognition and dementia. The original searches and the first update from 2020 have been described in the already published the systematic review and meta-analysis addressing PA and dementia [1].

After the first reduced update search, two additional extensive updates have been one: one targeting dementia in 2021 and one targeting cognition in 2022. The search terms for PA and study type in the extensive update targeting cognition carried out in 2022 are similar as in the already published update targeting dementia from 2021 [1]. The search terms for cognition are the same as in the original searches in 2017 to 2018. These additional extensive updates are better targeted than the first original search that yield greatly irrelevant studies. The searches of the latest update were done October 29, 20212 in CINAHL, PubMed, Web of Science, scopus, November 2, 2022 in PsycInfo and SPORTDiscus. Two examples of the original searches have been previously published [1]. Below are the 2022 update searches targeting PA and cognition from all databases.

© 2023 Iso-Markku P et al. *JAMA Network Open.*

eTable 1. The search strategy

Search terms	CINAHL [a] 29.10.2022	PubMed [b] 29.10.2022	Web of Science [c] 29.10.2022	Scopus [d] 29.10.2022	PsycInfo [e] 2.11.2022	SportDiscus [f] 2.11.2022
**1**	physical activity	physical activity	”physical activity”	”physical activity”	"physical activity".mp.	”aerobic exercise”
**2**	aerobic exercise	sport	”aerobic exercise”	”aerobic exercise”	"aerobic exercise".mp.	sport*
**3**	sport*	walking	sport*	sport*	walking.mp.	walking
**4**	walking	“physical training”	walking	walking	sport*.mp.	”physical activity”
**5**	physical training	“aerobic exercise”	”physical training”	“physical training”	"physical training".mp.	“physical training”
**6**	1 OR 2 OR 3 OR 4 OR 5	1 OR 2 OR 3 OR 4 OR 5	1 OR 2 OR 3 OR 4 OR 5	1 OR 2 OR 3 OR 4 OR 5	1 OR 2 OR 3 OR 4 OR 5	cognition
**7**	cognition	cognition	cognition	cognition	cognition.mp.	cognitive
**8**	cognitive	cognitive	cognitive	cognitive	cognitive.mp.	”executive function”
**9**	executive function	executive function	”executive function”	”executive function”	"executive function".mp.	TELE
**10**	TELE	TELE	TELE	TELE	TELE.mp.	TICS
**11**	TICS	TICS	TICS	TICS	TICS.mp.	MMSE
**12**	MMSE	MMSE	MMSE	MMSE	MMSE.mp.	3-MS
**13**	3-MS	3-MS	3-MS	3-MS	3-MS.mp.	”processing speed”
**14**	memory	memory	memory	memory	memory.mp.	”semantic fluency”
**15**	processing speed	processing speed	”processing speed”	”processing speed”	"processing speed".mp.	memory
**16**	verbal fluency	verbal fluency	”verbal fluency”	”verbal fluency”	"verbal fluency".mp.	”delayed recall”
**17**	semantic fluency	semantic fluency	”semantic fluency”	”semantic fluency”	“semantic fluency”.mp.	”verbal fluency”
**18**	reasoning	reasoning	reasoning	reasoning	reasoning.mp.	reasoning
**19**	delayed recall	delayed recall	”delayed recall”	”delayed recall”	“delayed recall”.mp.	prospective
**20**	7 OR 8 OR 9 OR 10 OR 11 OR 12 OR 13 OR 14 OR 15 OR 16 OR 17 OR 18 OR 19	7 OR 8 OR 9 OR 10 OR 11 OR 12 OR 13 OR 14 OR 15 OR 16 OR 17 OR 18 OR 19	7 OR 8 OR 9 OR 10 OR 11 OR 12 OR 13 OR 14 OR 15 OR 16 OR 17 OR 18 OR 19	7 OR 8 OR 9 OR 10 OR 11 OR 12 OR 13 OR 14 OR 15 OR 16 OR 17 OR 18 OR 19	7 OR 8 OR 9 OR 10 OR 11 OR 12 OR 13 OR 14 OR 15 OR 16 OR 17 OR 18 OR 19	longitudinal
**21**	prospective study	prospective	prospective	longitudinal	prospective.mp.	follow-up
**22**	longitudinal	longitudinal	longitudinal	follow-up	longitudinal.mp.	observational
**23**	follow-up	follow-up	follow-up	prospective	follow-up.mp.	cohort*
**24**	observational	“follow up”	observational	observational	"follow up".mp.	S1 OR S2 OR S3 OR S4 OR S5
**25**	cohort study	observational	cohort*	cohort	observational.mp.	S6 OR S7 OR S8 OR S9 OR S10 OR S11 OR S12 OR S13 OR S14 OR S15 OR S16 OR S17 OR S18
**26**	10 OR 11 OR 12 OR 13 OR 14	cohort	21 OR 22 OR 23 OR 24 OR 25	21 OR 22 OR 23 OR 24 OR 25	cohort*.mp.	S19 OR S20 OR S21 OR S22 OR S23
**27**	6 AND 9 AND 15	11 OR 12 OR 13 OR 14 OR 15 OR 16	6 AND 20 AND 26	6 AND 9 AND 16	21 OR 22 OR 23 OR 24 OR 25 OR 26	S24 AND S25 AND S26
**28**		6 AND 10 AND 17			6 AND 20 AND 27	

[a] Filters: English, Human, All adult, 12/2017 – 10/2022, Research articles, Search field: Abstract

© 2023 Iso-Markku P et al. *JAMA Network Open.*

[b] (((((((((((physical activity[Title/Abstract])) OR (sport[Title/Abstract])) OR (walking[Title/Abstract])) OR ("aerobic exercise"[Title/Abstract])) OR ("physical training"[Title/Abstract])) OR (physical activity[MeSH Terms])) OR (sport[MeSH Terms])) OR (walking[MeSH Terms])) OR (physical training[MeSH Terms])) OR (aerobic exercise[MeSH Terms])) AND (((((((((((((((((((cognition[Title/Abstract]) OR (cognition[MeSH Terms])) OR (cognitive[Title/Abstract])) OR (executive function[Title/Abstract])) OR (executive function[MeSH Terms])) OR (TELE[Title/Abstract])) OR (TICS[Title/Abstract])) OR (TICS[MeSH Terms])) OR (MMSE[Title/Abstract])) OR (MMSE[MeSH Terms])) OR (3-MS[Title/Abstract])) OR (memory[Title/Abstract])) OR (memory[MeSH Terms])) OR (processing speed[Title/Abstract])) OR (verbal fluency[Title/Abstract])) OR (semantic fluency[Title/Abstract])) OR (reasoning[Title/Abstract])) OR (delayed recall[Title/Abstract])) OR (delayed recall[MeSH Terms]))) AND (((((((((prospective[Title/Abstract]) OR (prospective[MeSH Terms])) OR (longitudinal[Title/Abstract]))) OR (follow-up[Title/Abstract])) OR ("follow up"[Title/Abstract])) OR (observational[Title/Abstract])) OR (cohort[Title/Abstract])) OR (cohort[MeSH Terms]))

Filters applied: Adult: 19+ years, English, from 2017/12/14 - 2022/10/29, Field of search: Title/Abstract, MeSH-terms

[c] Filters: Review Article (Exclude – Document Types) and 2022 or 2021 or 2020 or 2019 or 2018 or 2017 (Publication Years) and Article (Document Types) and Pediatrics or Orthopedics or Cardiovascular System Cardiology or Research Experimental Medicine or Nutrition Dietetics or Science Technology Other Topics or Mathematics or Endocrinology Metabolism or Biochemistry Molecular Biology or Surgery or Education Educational Research (Exclude – Research Areas) and Tropical Medicine or Social Work or Parasitology or Operations Research Management Science or Music or Meteorology Atmospheric Sciences or Mechanics or International Relations or Government Law or Geology or Forestry or Fisheries or Film Radio Television or Entomology or Construction Building Technology or Chemistry or Biotechnology Applied Microbiology or Biodiversity Conservation or Veterinary Sciences or Religion or Plant Sciences or Microbiology or Philosophy or Medical Ethics or Imaging Science Photographic Technology or History or Hematology or Criminology Penology or Anthropology or Zoology or Virology or Transportation or Instruments Instrumentation or Automation Control Systems or Women Apos S Studies or Urban Studies or Substance Abuse or Rheumatology or Public Administration or Linguistics or Geography or Arts Humanities Other Topics or Women S Studies or Robotics or Food Science Technology or Cell Biology or Biophysics or Audiology Speech Language Pathology or Anesthesiology or Dentistry Oral Surgery Medicine or Critical Care Medicine or Agriculture or Ophthalmology or Otorhinolaryngology or Information Science Library Science or Urology Nephrology or Ethnic Studies or Dermatology or Emergency Medicine or Obstetrics Gynecology or Reproductive Biology or Mathematical Computational Biology or Gastroenterology Hepatology or Toxicology or Telecommunications or Engineering or Business Economics or Respiratory System or Infectious Diseases or Oncology or Pharmacology Pharmacy or Communication (Exclude – Research Areas) and SPORTS CONCUSSION VIRTUAL CONFERENCE or 27TH ANNUAL MEETING OF THE SOCIETY FOR THE STUDY OF INGESTIVE BEHAVIOR SSIB (Exclude – Conferences/Meeting Titles) and English (Languages)

Field of search: Abstract

[d] Filters: English, Publication source: Journal, Published: 2017 -2022 (search 29.10.2021), Field of search: Abstract

Search: ((ABS ("physical activity")) OR (ABS ("aerobic exercise")) OR (ABS (sport*)) OR (ABS (walking)) OR (ABS ("physical training"))) AND ((ABS (cognition)) OR (ABS (cognitive)) OR (ABS ("executive function")) OR (ABS (tele)) OR (ABS (tics)) OR (ABS (mmse)) OR (ABS (3-ms)) OR (ABS (memory)) OR (ABS ("processing speed")) OR (ABS ("verbal fluency")) OR (ABS ("semantic fluency")) OR (ABS (reasoning)) OR (ABS ("delayed recall"))) AND ((ABS (prospective)) OR (ABS (longitudinal)) OR (ABS (follow-up)) OR (ABS (observational)) OR (ABS (cohort))) AND (LIMIT-TO (DOCTYPE , "ar")) AND (EXCLUDE (SUBJAREA , "ENGI") OR EXCLUDE (SUBJAREA , "PHAR") OR EXCLUDE (SUBJAREA , "COMP") OR EXCLUDE (SUBJAREA , "IMMU") OR EXCLUDE (SUBJAREA , "BUSI") OR EXCLUDE (SUBJAREA , "DENT") OR EXCLUDE (SUBJAREA , "EART") OR EXCLUDE (SUBJAREA , "ECON") OR EXCLUDE (SUBJAREA , "VETE") OR EXCLUDE (SUBJAREA , "CHEM") OR EXCLUDE (SUBJAREA , "ENER") OR EXCLUDE (SUBJAREA , "DECI") OR EXCLUDE (SUBJAREA , "PHYS") OR EXCLUDE (SUBJAREA , "CENG") OR EXCLUDE (SUBJAREA , "MATE") OR EXCLUDE (SUBJAREA , "MATH")) AND (LIMIT-TO (PUBYEAR , 2022) OR LIMIT-TO (PUBYEAR , 2021) OR LIMIT-TO (PUBYEAR , 2020) OR LIMIT-TO (PUBYEAR , 2019) OR LIMIT-TO (PUBYEAR , 2018) OR LIMIT-TO (PUBYEAR , 2017)) AND (LIMIT-TO (LANGUAGE , "English")) AND (EXCLUDE (EXACTKEYWORD , "Controlled Study") OR EXCLUDE (EXACTKEYWORD , "Randomized Controlled Trial") OR EXCLUDE (EXACTKEYWORD , "Cross-sectional Study") OR EXCLUDE (EXACTKEYWORD , "Adolescent") OR EXCLUDE (EXACTKEYWORD , "Child") OR EXCLUDE (EXACTKEYWORD , "Cross-Sectional Studies") OR EXCLUDE (EXACTKEYWORD , "Clinical Trial") OR EXCLUDE (EXACTKEYWORD , "Health Behavior") OR EXCLUDE (EXACTKEYWORD , "Preschool Child") OR EXCLUDE (EXACTKEYWORD , "Randomized Controlled Trial (topic)"))

[e] Limits: (("300 adulthood <age 18 yrs and older>" or 320 young adulthood <age 18 to 29 yrs> or 340 thirties <age 30 to 39 yrs> or 360 middle age <age 40 to 64 yrs> or "380 aged <age 65 yrs and older>" or "390 very old <age 85 yrs and older>") and "0110 peer-reviewed journal" and journal article and english and human and yr="2017 - 2022"), Search field: Abstracts

[f] Limiters: Peer reviewed, Date 20171201-; Field of search: Abstract

© 2023 Iso-Markku P et al. *JAMA Network Open.*

© 2023 Iso-Markku P et al. *JAMA Network Open.*

1.2 Deviations from the original study plan

1. We report the results in two parts
    - The numbers of sub-analyses grew so large that we decided to split the meta-analysis into two parts for clarity. The other part on dementia has already been published [1].

2. Our aim was to study what kind of physical activity (PA) is associated with cognitive decline

3. The method of measuring education was extracted with precision but was not examined with a separate sub-analysis because of vast heterogeneity in the classifications.

4. The quality assessment tool was developed only after research plan was ready and adjusting for chronic diseases at baseline was not included because adjusting for education and some vascular risk factor was deemed more important.

5. We did not use Review Manager 5 but Covidence for handling data.

6. We conducted the prespecified moderator analyses but the classifications varied a little according to the data found and continuous variables we assessed with meta-regression employing the full statistical power.

7. We did not perform separate analyses for different genders because there was not enough data: almost all studies presented results only for men and women jointly and very few studies reported the results for gender interaction test.

8. There was not enough data for a separate analysis for twin studies.

9. Originally, we aimed to include only studies that had a valid measure of baseline cognition. The studies, however, used this information differently and the sensitivity analysis of comparing studies adjusting for baseline cognition or not was added only afterwards.

10. All other sensitivity analyses were prespecified except for the analyses of cognition measure and adjustment for other vascular risk factors.

1.3 Additional specifications to the inclusion and exclusion criteria, data extraction, and quality review

Specifications to the inclusion and exclusion criteria at the title and abstract screening phase in cases of disagreements:

The studies are included if

- All the other criteria are fulfilled but cognition is the independent variable and PA the dependent variable. These we include because in the full text, researchers may consider the association vice versa also.
- It is not clear whether the article describes cross-sectional or longitudinal results from a longitudinal study (this has not been specified and for example, the cohort's name encompasses the word longitudinal or something similar).
- It is unclear at this point whether cognition is measured or self-reported.
- The study adjusts for vascular risk factors and on the basis of the abstract, it is not absolutely clear, what these vascular risk factors are (PA might be one of them).

The studies are excluded if

- Outcome variable is clearly something else than cognition.
- It has been specified in the abstract that the report is cross-sectional even if "longitudinal" or similar phrase is in the name of the study cohort.

Specifications to the inclusion and exclusion criteria at the full-text review phase in cases of disagreements:

The studies are included if

© 2023 Iso-Markku P et al. *JAMA Network Open.*

- We have to have either a measure of whole cognition based on which it is possible to deduce if the participant has cognitive impairment or not at baseline or we have a valid measure of a single cognitive domain and it has been measured both at baseline and at follow-up and the analyses take into account the baseline measure of cognition

The studies are excluded if

- Only figure of the estimate of the association of PA and cognition has been presented but no numerical results and the authors do not provide these numbers after email inquiry.
- The outcome is a subjective rating of one's own memory.
- A method to screen dementia at baseline has been a measure of dementia difficulty not a valid measure of cognition (like for example Clinical Dementia Rating).
- The study examines the association of only tai chi, pilates, yoga or other similar types of low-intensity PA while ignoring all other PA.
- Studies measuring only indoor PA with PIR sensors.
- The study uses linear mixed model to examine the association of PA with cognition at all measurement points including the baseline
- The study examines the association of PA intercept (from a growth model or latent intercept), not baseline PA.

Data extraction:

The studies were deemed to adjust for preceding level of cognition if they had a valid measure of preceding cognition as covariate in their analysis model. An exception to this was linear mixed models with the level of cognition as the outcome in which the study needed to have baseline cognition x time interaction term in the model.

The estimates with the best quality score and the most extensive set of adjustments were extracted. If two sets of results were presented, we chose the unimputed ones with cognitive impairment at baseline excluded, dementia developed during the first years of follow-up excluded, or more sensitive cut-off for cognitive impairment. For example, if the main analysis included participants with existing cognitive impairment but a subgroup analysis with a smaller sample size excluded those with existing cognitive impairment, the estimate of association from the subgroup analysis was included in the meta-analysis.

One study presented the estimates of the association between PA with episodic memory and processing speed in different age groups [2]. We only used the estimates for middle-age to avoid baseline levels being lowered due to dementia. Some of the studies had reported only unstandardized beta coefficients without standard deviations of the outcome variable or it was unclear for us whether the estimate was standardized or not. The authors were contacted to obtain the standard deviation of the outcome variable in these cases: many replied and cordially provided us with the information while some did not.

Quality review:

One criterion of the quality assessment tool presented in our previous meta-analysis of PA [1] and dementia was different for this meta-analysis of PA and cognition: the length of follow-up had to be at least 10 years for all studies except for those in which the follow-up was in midlife (defined as mean or median age less than 55 years and maximum age less than 65 years or mean age plus one standard deviation (SD) less than 60 years). For studies in which follow-up was in midlife, good quality could be reached with at least five years of follow-up.

If a quality criterion could not be assessed from the study article, at maximum three cited publications were reviewed.

1.3 More details about the transformations used in the meta-analyses

1.3.1 Binary analyses

In the analysis of dichotomous outcomes, odds ratios and hazard ratios were transformed into risk ratios. Odds ratios were transformed with the following formula when the outcome was rare (< 10 %) [3].

© 2023 Iso-Markku P et al. *JAMA Network Open.*

$$RR = \frac{OR}{(1-p0+p0*OR)} \quad (1)$$

Where:
p0= outcome incidence in the whole study population
OR=Odds ratio

If the outcome was common (> 10 %), we used square root transformation of odds ratios [4]:

$$RR = \sqrt{OR} \quad (2)$$

Where:
RR=Risk ratio
OR=Odds ratio

Hazard ratios were transformed using the following formula dementia for the reference group) [5].

$$RR = \frac{1-e^{HR*\ln(1-r)}}{r} \quad (3)$$

Where:
r=The incidence rate of cognitive impairment or decline
RR=Risk ratio
HR=Hazard ratio

If the number of persons with and without event at follow-up were given, risk ratio was calculated with the following formula:

$$RR = \frac{\frac{N_{Physically\ active\ who\ became\ cognitively\ impaired}}{N_{All\ physically\ active}}}{\frac{N_{Physically\ inactive\ who\ became\ cognitively\ impaired}}{N_{All\ physically\ inactive}}} \quad (4)$$

Where:
N=Sample size
RR=Risk ratio

If confidence interval for a binary outcome was not given, but a p-value for it was presented, we calculated the confidence interval from the p-value in the following manner [6]:

$$z = -0.862 + \sqrt{(0.743 - 2.404 * \ln(p\ value)} \quad (5)$$

$$SE = \frac{\ln(binary\ outcome)}{z} \quad (6)$$

$$Upper\ limit\ of\ 95\ \%\ CI = e^{(\ln(binary\ outcome)+1.96*SE} \quad (7)$$

$$Lower\ limit\ of\ 95\ \%\ CI = e^{(\ln(binary\ outcome)-1.96*SE)} \quad (8)$$

Where:
SE= standard error
z = z -score
CI = Confidence interval

© 2023 Iso-Markku P et al. *JAMA Network Open.*

Most studies reported hazard, odds or risk ratio for the risk of cognitive impairment or decline, but a few studies reported risks for different cognitive trajectories. In almost all studies examining risks for different cognitive trajectories, only one of these trajectories was clearly declining and the other ones were relatively stable [7 – 10]. We pooled the risk for rapid decline in comparison with the most stable cognition group into the meta-analysis. Any trajectories between the most and least stable were excluded from this meta-analysis. In one study, two trajectories were declining and one was stable [11]. Since, the other declining trajectory had the same starting point as the stable group, we included the risk for this declining trajectory into the meta-analysis.

1.3.2 Analyses of rate of change in cognition

In many papers, the outcome was the rate of change in cognition (for example, the interaction term of PA x time from linear mixed models). The time unit used in all but one study was years. One study presented their results per five years[12] and these were divided by five to yield the rate of change per year. To pool the studies of rate of change, we multiplied the standardized regression coefficients for rate of change with the length of the follow-up in years to yield us the change in cognition [13].

1.3.3 Estimation of a standard deviation for a continuous outcome variable

If standard deviations were reported only for subgroups but not for the whole group we used the following formula to derive the within-sub-group standard deviation [14]:

$$SD_{full\ sample} = \sqrt{\frac{(n_1-1)*SD_1^2+(n_2-1)*SD_2^2+\frac{n_1 n_2}{n_1+n_2}*(M_1-M_2)^2}{n_1+n_2-1}} \quad (9)$$

Where:
$SD_{full\ sample}$ = Standard deviation of the outcome of the full sample
SD_1 = Standard deviation of the outcome for the group 1
SD_2 = Standard deviation of the outcome for the group 2
n_1 = Sample size of the group 1
n_2 = Sample size of the group 2
M_1 = Mean for the group 1
M_2 = Mean for the group 2

When only standard error was given for the outcome, we transformed the standard error to standard deviation with the following formula:

$$SD = SE * \sqrt{N} \quad (10)$$

Where:
SD = Standard deviation of the outcome
SE = Standard error of the outcome
N = Sample size

When we did not have standard deviation, standard error or confidence interval of the outcome at baseline, we approximated it from the minimum and maximum value in the following manner [14]:

$$SD = \frac{(maximum-minimum)}{6} \quad (11)$$

Where:

© 2023 Iso-Markku P et al. *JAMA Network Open.*

SD= Standard deviation

When we did not have information on standard deviation of cognition at baseline, but standard deviation for intercept of cognition from the model of cognition change was given, we used the standard deviation of the intercept [15].

When standard deviation was not given in the article, we used interquartile range to estimate the standard deviation [14]:

$$SD = \frac{(upper\ quartile - lower\ quartile)}{1.349} \quad (12)$$

Where:
SD=Standard deviation

For animal fluency (the number of animal names produced in 60 seconds), we used the interquartile range from other published data on noninstitutionalized population of older adults as a reference [16].

When a ln transformation of a variable was used because of a skewed distribution, we calculated a standard deviation for the ln transformed variable in the following manner [14]:

$$SD(lnX) = \sqrt{\ln\left(1 + \frac{SD_X^2}{M_X^2}\right)} \quad (13)$$

Where:
SD(lnX)=Standard deviation of a ln transformed PA variable
SD_X=Standard deviation of PA variable (not ln transformed)
M_X=Mean of PA variable

For the studies in which we were not able to standardize the outcome, we contacted authors to provide us with the standard deviation of the outcome.

1.3.4 Standardized regression coefficient from comparison of mean values between two exposure groups

When regression coefficients or post-follow-up mean scores with standard deviations for a binary PA variable were presented we calculated standardized regression coefficient with the following formula [14]:

$$\beta = \sqrt{\frac{n_1 n_2}{(n_1+n_2)^2}} * \frac{(M_1 - M_2)}{SD(Y)} = \sqrt{\frac{n_1 n_2}{(n_1+n_2)^2}} * \frac{b}{SD(Y)} \quad (14)$$

Where:
β = Standardized regression coefficient
n_1 = Sample size of the group 1
n_2 = Sample size of the group 2
M_1 = Mean for the group 1
M_2 = Mean for the group 2
SD(Y) = full sample standard deviation of outcome variable Y
b = unstandardized regression coefficient.

Confidence interval for the results was calculated from the p-value presented in the article in the following manner [6]:

$$z = -0.862 + \sqrt{(0.743 - 2.404 * \ln(p\ value)} \quad (5)$$

$$SE = \frac{\beta}{z} \quad (15)$$

$$Upper\ limit\ of\ 95\ \%\ CI = \beta + 1.96 * SE \quad (16)$$

$$Lower\ limit\ of\ 95\ CI = \beta - 1.96 * SE \quad (17)$$

Where:
SE= standard error
z = z -score
β = Standardized regression coefficient
CI = Confidence interval

If p-value was presented as "< 0.05", we transformed it into confidence intervals using an approximation of p-value = 0.049 in the calculations and similarly, if p-value was denoted as "> 0.1" or ">0.05", we approximated it to p-value ≈0.55.

If a p-value was not given, we used the following formula to estimate standard error for β [14] and calculated confidence interval with equations 10 and 11:

$$SE(\beta) = \frac{SD(X)}{SD(Y)} SE(b) = \frac{SD(X)}{SD(Y)} SE(M_1 - M_2) = \sqrt{\frac{n_1 n_2}{(n_1+n_2)^2}} * \frac{SE(b)}{SD(Y)} \quad (18)$$

$$SE(M_1 - M_2) = \sqrt{\frac{(n_1-1)SD_1^2+(n_2-1)SD_2^2}{n_1+n_2-2} * \sqrt{\frac{1}{n_1}+\frac{1}{n_2}}} \quad (19)$$

Where:
SE(β) = Standard error of standardized regression coefficient
n_1 = Sample size of the group 1
n_2 = Sample size of the group 2
SD(Y) = full sample standard deviation of outcome variable Y
SD(X) = Standard deviation of independent variable X
SE (b)= Standard error of unstandardized regression coefficient.
M_1 = Mean for the group 1
M_2 = Mean for the group 2
SD_1 = Standard deviation of the outcome for the group 1
SD_2 = Standard deviation of the outcome for the group 2

1.3.5 Standardized regression coefficient for change from comparison of pre- and post- follow-up test scores between two exposure groups

When regression coefficients for change or pre- and post-follow-up mean scores with standard deviations for a binary PA variable were presented we calculated standardized regression coefficient with the following formula [14]:

$$\beta = \sqrt{\frac{n_1 n_2}{(n_1+n_2)^2}} * \frac{(Change_1 - Change_2)}{SD(Y)} = \sqrt{\frac{n_1 n_2}{(n_1+n_2)^2}} * \frac{b_{Change}}{SD(Y)} \quad (20)$$

Where:
β = Standardized regression coefficient
n_1 = Sample size of the group 1
n_2 = Sample size of the group 2
$Change_1$ = Mean change score for the group 1
$Change_2$ = Mean change score for the group 2
SD(Y) = full sample standard deviation of outcome variable Y at baseline [13, 17]

© 2023 Iso-Markku P et al. *JAMA Network Open.*

b = unstandardized regression coefficient.

Confidence interval for the results was calculated from the p-value with formulas 4, 15, 16 and 17. If p-value was not given but a F-value from repeated measures of analysis of variance (ANOVA) was given, we calculated the p-value from the F-value.

If a p-value for the unstandardized regression coefficient was not given, we used the following formula to estimate standard error for standardized regression coefficient [14] and calculated confidence interval with equations 16 and 17:

$$SE(\beta) = \frac{SD(X)}{SD(Y)}\,SE(b) = \frac{SD(X)}{SD(Y)}SE(Change_1 - Change_2) = \sqrt{\frac{n_1 n_2}{(n_1+n_2)^2}} * \frac{SE(b)}{SD(Y)} \quad (21)$$

$$SE(Change_1 - Change_2) = \sqrt{\frac{(n_1-1)SD_1^2+(n_2-1)SD_2^2}{n_1+n_2-2}} * \sqrt{\frac{1}{n_1}+\frac{1}{n_2}} \quad (22)$$

Where:
SE(β) = Standard error of standardized regression coefficient
n_1 = Sample size of the group 1
n_2 = Sample size of the group 2
SD(Y) = full sample standard deviation of outcome variable Y at baseline
SD(X) = Standard deviation of independent variable X
SE (b)= Standard error of unstandardized regression coefficient.
$Change_1$ = Change for the group 1
$Change_2$ = Change for the group 2
SD_1 = Standard deviation of the outcome at baseline for the group 1
SD_2 = Standard deviation of the outcome at baseline for the group 2

1.3.6 Standardized regression coefficient from more than two exposure groups

We used linear trend test to estimate standardized regression coefficient when the outcome was presented for a categorized exploratory variable with more than two exposure groups. A simple linear regression line was drawn with categories of exposure as x-axis (contrast values) and the outcome values as the y-axis [14]. We used MET (metabolic equivalent of task) -minutes, absolute energy expenditure, or PA scores as contrast values if readily available or easily calculated and values 0, 1, 2, 3, … k in cases where MET-minutes, absolute energy expenditure, or PA scores was not easily available (k is the number of categories). β with standard error was obtained in the following manner [14]:

$$\beta = \frac{SD(contrast)}{SD(Y)} b_c \quad (23)$$

$$SE(\beta) = \frac{SD(contrast)}{SD(Y)} SE(b_c) \quad (24)$$

Where:
β = Standardized regression coefficient
SD(contrast) = Standard deviation of the selected contrast values
SD(Y) = Standard deviation of the outcome variable
b_c = Regression coefficient of the linear regression line from the linear trend test
SE(β) = Standard error of the standardized regression coefficient
$SE(b_c)$ = Standard error of the regression coefficient from the linear trend test.

One study reported follow-up scores with standard deviations and p-value from a generalized linear regression model but not the regression coefficient. For this study, we calculated the standardized regression coefficient from follow-up scores using formulas 23 and 8. We calculated confidence intervals for this standardized regression coefficient using the p-value from the generalized linear regression model with formulas 4, 15, 16 and 17.

© 2023 Iso-Markku P et al. *JAMA Network Open.*

1.3.7 Dose-response plots

We approximated the midpoint of each PA category to metabolic equivalent of energy expenditure (MET) -minutes [1, 18]. Next, we plotted these MET-minutes for each category against the relevant magnitude of association of the corresponding category in a scatterplot of MET-minutes and magnitudes of association for cognition. The scatter dot sizes were drawn to reflect the sample size of the corresponding PA category. After this, we fitted linear, polynomial, and quadratic lines to these scatter plots and chose the visually best one to be presented in the graphs. The studies that used continuous measures of PA were excluded from this analysis.

The midpoint of each PA category was regarded as the PA exposure for each group [18]. Some studies reported PA in MET-minutes or MET-hours. These studies did not need approximation. Some studies reported the amount of PA and some description of the intensity. For these studies, the intensity was estimated in the following manner: walking = 3.5 MET, sport = 4 MET, moderate PA = 4.5 MET, vigorous PA = 8 MET, light PA = 2.25 MET, light and moderate PA combined = 3.25 MET (midpoint of 4 (sport) and 2.5 (light PA)), moderate- to vigorous PA = 5 MET. For studies in which PA was assessed with frequency but not with duration, the duration was estimated to be 45 minutes for moderate- to vigorous PA and 60 minutes for other PA. Studies in which PA was expressed in kcal, MET-minutes were calculated using the following formula [1]:

$$MET - minutes = \frac{60*kcal}{kg} \qquad (25)$$

Where:
kcal=Kilocalorie
kg=Body weight in kilograms

When mean body weights were not given in the study, a continental average body weights were used [19]. As in our previous meta-analysis [1], we imputed the PA exposure for studies in which MET-minutes was impossible to estimate from other similar studies. The cut-off for meeting the PA recommendation [20] of moderate- to vigorous PA was estimated to be 500 MET-minutes per week. If a mean could not be calculated for the highest PA category, the mean for the highest PA category group was set near the cut-off.

1.3.8 Other approximations used in the meta-analysis

Most studies reported mean or median baseline age but for studies that did not, the midpoint of the range given was used.

1.4 Tests to measure cognition in the meta-analysis

© 2023 Iso-Markku P et al. *JAMA Network Open.*

eTable 2. Definitions of cognitive impairment and decline

Publication	Definition of cognitive impairment
Brunner 2017	MMSE score is less than 27
Clark 2016	MMSE score is 23 or less
de Frias 2014	Scored at least 1.5 SD below the group mean on one or more tests (WAIS-R DSST, the Letter Series test, immediate recall, the Controlled Associations test from the Educational Testing Service kit of factor-referenced cognitive tests, A recognition vocabulary test).
Elwood 2013	Score on CAMCOG of less than 83, or a decline in CAMCOG score of 10 or more since the earlier cognitive test
Etgen 2010	The Blessed Information Memory Concentration Scale: score higher than 7
Gao 2017	Chinese MMSE below 18
Ho 2001	The Clifton Assessment Procedure for the elderly (CAPE) information/orientation part (12 questions): 7 or lower
Hughes 2015	CDR stage of 0.5
Infurna 2016	TICS: A score of 8 or less (out of 35 points)
Iso-Markku 2016	TELE: a telephone assessment of dementia ≤ 17.5
Kim 2011	Cognitive decline: decline in MMSE score of at least three points from baseline to follow-up (i.e. >1 SD below the mean change in scores)
Laurin 2001	1) 3MS (at least 78 scores were asked for 3-stage clinical evaluation) 2) nurse evaluation 3) physician evaluation 4) neuropsychological test battery 5) consensus diagnosis of CIND (cognitive impairment -no dementia)
Lipnicki 2017	1) self or informant complaint of memory or other cognitive function decline, 2) objective cognitive impairment (at least one test score ≥ 1.5 SD below published normative values in a broad neuropsychological battery, adjusted for age and/or education where possible) 3) no dementia; and no or minimal impairment in instrumental activities of daily living attributable to cognitive impairment
Newman 2009	A score of less than 80/100 on the 3MSE. For individuals without an in-person examination, a Telephone Interview for Cognitive Status (TICS) was obtained and for those with a proxy interview, the Informant Questionnaire for Cognitive Decline in the Elderly (IQCODE) was administered. Based on a prior validation study, estimated 3MSE scores were calculated based on these tests to classify current level of cognitive function.
Stewart 2003	Orientation (MMSE), immediate and delayed verbal recall and delayed word list recognition (CERAD, immediate recall defined as maximum recall over three presentations), and visual attention and motor speed (Trail A test). A composite measure of cognitive change was derived through factor analysis. Participants in the lowest quintile were defined as having cognitive decline.
Sumic 2007	Repeated abnormal scores on the MMSE (< 24) or the CDR (= 0.5) on two consecutive assessments.
Verdelho 2012	The neuropsychological battery included the MMSE as a global measure of cognitive function; the Vascular Dementia Assessment Scale cognitive subscale (VADAS-Cog) as a comprehensive instrument to assess orientation, language, ideational and constructional praxis, immediate memory and delayed recall, attention and speed of mental processing; and the Stroop and Trail Making tests as measures of executive. Patient cognitive status was classified into the following groups: (1) dementia;

© 2023 Iso-Markku P et al. *JAMA Network Open.*

	(2) cognitive impairment not dementia; or (3) no cognitive impairment. The risk for both dementia and cognitive impairment not dementia was extracted for this meta-analysis.
Woodard 2012	Significant cognitive decline was defined as exhibiting a one SD reduction or greater on at least one of the three principal outcome indices (Mattis Dementia Rating Scale -2, RAVLT Sum of Trials 1-5, RAVLT Delayed Recall). Standardized residual change scores were computed to adjust for baseline performance, practice effects, and regression to the mean.
Zhu 2017	Six Item Screener score <4 (range 0 to 6)
Verghese 2009	Participants were diagnosed with VCI (from mild vascular cognitive impairment to vascular dementia) if they met the following 2 criteria:<br>1. Nonamnestic impairment. Digit Symbol Substitution, Digit span (total span), and Category Fluency tests. Raw scores on each of these tests administered at baseline in the entire cohort were converted to standardized z scores, which were averaged to form a composite score. Nonamnestic impairment was defined as performance at or below 1 standard deviation from the mean composite score in this cohort.<br>2. Cerebrovascular disease defined as presence of any 1 of Hachinski ischemic score of 4 or higher, presence of hemiparesis on clinical evaluation, or history of strokes verified by medical records and imaging studies.
Verghese 2006	Amnestic mild cognitive impairment (aMCI). Subjects were diagnosed with aMCI if they met the following criteria: 1) does not meet criteria for dementia; 2) objective memory impairment defined as three or more errors on the five-item Blessed test memory phrase. This cutscore corresponds to performance at or below 1.5 SDs from the mean (1.1 ± 1.4) in this age restricted sample.
Wang 2006	Neuropsychological evaluation: the cognitive status was assessed using the Chinese version of MMSE with the cutpoint previously defined as 17 (illiteracy), 20 (6 years of education), and 24 (6 years of education) for cognitive impairment (below cut-off).
Beauchet 2020	3MS (≤79/100)
Ramoo 2022	Malaysian MMSE < 25 for uneducated, < 27 for individuals with primary education and < 29 for individuals with secondary or above education
Strozza 2020	MMSE 0-23.
Dupré 2020	MoCA < 27
Thompson 2022	KICA-cog (Kimberley Indigenous Cognitive Assessment) and comprehensive health assessment. A consensus diagnosis: incidence of dementia or CIND.
He 2021	MMSE: cut-off 17/18 for those with no education, 20/21 for those with primary education only, and 24/25 for those with education beyond the primary level
Shih 2017	The3MSE and a delayed word recall trial from the Spanish English Verbal Learning Test (SEVLT). Participants were referred for a neuropsychological test battery and a standard neuropsychological examination (Informant Questionnaire on Cognitive Decline in the Elderly (IQCODE)) by a geriatrician if 1) their baseline scores on 3MSE or SEVLT fell below the 20th percentile, or 2) had ≥ 8- point decreased on the 3MSE or ≥ 3-point decreased SEVLT and the scores below the 20th percentile at follow-up. A team of neurologists and a neuropsychologist reviewed all referred cases and classified them as demented, CIND, or cognitively normal on the basis of neuropsychological test battery and IQCODE as well as their history, mental status examination, and findings from the neurologic examination when available. We combined dementia and CIND partly to improve our statistical power.

© 2023 Iso-Markku P et al. *JAMA Network Open.*

Krell-Roesch 2021 German J	MCI: (1) cognitive concern expressed by a physician, informant, participant, or study coordinator; (2) impairment in one or more cognitive domains (memory, attention/executive function, language, or visuospatial skills); (3) essentially normal functional activities; and (4) absence of dementia. Participants with MCI had a CDR score of 0 or 0.5; however, the final diagnosis of MCI was based on all available data. Cognitive domains were assessed with the following neuropsychological test battery: memory (delayed recall trials from Rey Auditory Verbal Learning Test, Wechsler Memory Scale Revised Logical Memory and Visual Reproduction subtests);language (Boston Naming Test, category fluency); visuospatial skills (WAIS-R Picture Completion and Block Design subtests); and attention/executive function (Trail-Making Test Part B, WAIcS-R, Digit Symbol Substitution subtest).
	Definition of cognitive decline
Hildreth 2014	A decrease of 2 points at follow up in MMSE
Iwasa 2012	Change in MMSE score -3 or more
Lee 2013	A decline of at least three points in MMSE score from baseline to follow-up
Leung 2011	A one-point drop in z-score in the Chinese MMSE. The z-score was derived from the raw scores based of participants who had a global CDR of zero at the baseline.
Lytle 2004	MMSE decline 3 points
Middleton 2011	A decline of at least one standard deviation (9 points) in 3MS
Niti 2008	A decline of one or more points in the MMSE
Pignatti 2002	A loss of one or more points in MSQ
Yaffe 2001	A decrease of 3 or more points in modified MMSE
Pitrou 2022	Cognitive decline is over 3 points decrease in MMSE
	Cognitive trajectories
Min 2018[1]	MMSE trajectory (sharp declining)
Chen 2016	SPMSQ trajectory: starting high and declining
de Looze 2022	Cognitive trajectory low-declining based on the following tests: 10-word immediate recall (2 trials), 10-word delayed recall, semantic verbal fuency (animal naming), and orientation (date, day, month, year).
Fassier 2022	Global score is the average of the z-scores of TICS, immediate and delayed recalls of the East Boston Memory Test, delayed recall of TICS 10-word list, test of category fluency and digit backwards test. Three trajectories were constructed and only one trajectory declined (other ones were high stable and medium stable).
McGarrigle 2022	MMSE trajectory 'Moderate decline'

Abbreviations: 3MS = Modified Mini Mental State Examination, CAMCOG = Cambridge Cognitive Examination, CDR = Clinical Dementia Rating, DSST = Digit Symbol Substitution Test, MMSE = Mini Mental State Examination, MOCA = Montreal Cognitive Assessment, MSQ= Mental Status Questionnaire, RAVLT = Rey Auditory Verbal Learning Test, SD=Standard deviation, SPMSQ = Short Portable Mental Status Questionnaire, WAIS-R = Wechsler Adult Intelligence Scale Revised

© 2023 Iso-Markku P et al. *JAMA Network Open.*

eTable 3. Tests used to assess continuous global cognition, verbal fluency and naming, working memory, verbal ability and visuo-spatial ability

Abbreviations: 3MS = Modified Mini Mental States Examination, CANTAB = Cambridge Neuropsychological Test Automated Battery, MMSE = Mini Mental State Examination, TICS =

Global cognition	Verbal fluency and naming	Working memory	Verbal ability	Visuo-spatial ability
A summary score of neuropsychological battery consisting many tests of multiple specific cognitive domains	Phonemic fluency	CANTAB Spatial Working Memory	WAIS Vocabulary: Provide definitions of words., Picture Vocabulary: Name the pictured object., Antonym Vocabulary: Select the best antonym of the target word. Synonym Vocabulary: Select the best synonym of the target word.	WAIS-R: Picture Completion and Block Design subtests
MMSE	Category fluency	Total number of correct items of the forward and backward scores of the WAIS digit span	Vocabulary test (20 words, each of which had another five words next to it. For each 20 words, the participant was asked to select which of the five words next to it had a similar meaning)	CANTAB Rapid Visual Information Processing
Partial MMSE	Boston Naming Test, Verbal Fluency, and a 15-item reading test	Digit Span Forward and Digit Span Backward and Digit Ordering	A 30-item vocabulary task, where participants were instructed to underline the word representing the synonym of each target word.	a 15-item version of Judgment of Line Orientation and a 16-item version of Standard Progressive Matrices
At least 10 tests of Wechsler Adult Intelligence Scale	Boston Naming Tests, category fluency, phonemic fluency			Abstract matching 1 and 2, distance judgement, length judgement, mental rotation, visual search, shape judgement
	Boston Naming Tests and category fluency			
TICS	Fluency A: recall words with initial letter A. Fluency M: generate words that contain exactly five letters and have initial letter M. Fluency B: recall occupations with initial letter B			
Modified TICS	Boston Naming Tests and category fluency			
3MS				
Short Portable Mental Status Questionnaire				

Telephone Interview for Cognitive Status, WAIS = Wechsler Adult Intelligence Scale, WAIS-R= Wechsler Adult Intelligence Scale Revised

© 2023 Iso-Markku P et al. *JAMA Network Open.*

eTable 4. Tests used to assess episodic memory, executive function and processing speed

Executive function	Processing speed	Episodic memory
Trails Making Test B (adjusted with part A)	Digit Symbol Substitution Test	CVLT immediate and delayed recall
Alice Heim 4-I	The Symbol Digit Modalities Test	HVLT immediate and delayed recall
IU Token Test	Number Comparison	RVLT immediate and delayed recall
Stroop interference	Pattern Comparison	Immediate recall, delayed recall and recognition of 10-word list from CERAD
Digits backwards, a shortened version of CANTAB Spatial Working Memory Tests and Stroop Word-Color Interference	Stroop part 1 (word reading) and part 2 (color naming)	East Boston Memory Test immediate and delayed recall
Animal fluency and a letter cancellation test (speed and accuracy)	Trails Making Test Part A	TICS 10-word list recall
The symbol Digit Modalities Test, Stroop interference, animal fluency and Trails Making Tests B – A	Digit cancellation	Delayed recall of the Rey-Osterrieth Complex figure test
Digit span backwards, Trails Making Test A and B, Letter Digit Substitution Test, Verbal fluency	Maze subtask of VADAS	Logical Memory Test
The difference in time to complete the Color Trails test Form 1 and Form 2 and the sum of the Odd-Man-Out subtests 2 and 4	the Grooved Pegboard task in the nondominant hand, the Color Trails test Form 1, and the Visual-Motor Integration test	Paired Associates
Behavioral dyscontrol scale	CANTAB Reaction time	IU Story Recall
Visual Elevator test, Brixton Spatial Anticipation test and Verbal Fluency test		Wecshler Memory Scale revised Logical Memory and Visual Reproduction
Backward digit span, category fluency, number series, backward counting, stop-go switch task		Action recall, sentence recall, cued recall, word list learning from Betula study [21]
Stroop interference, Letter Digit Substitution Task, category fluency		
Trails Making Tests B and Digit Symbol Substitution Test		Other immediate and delayed recall tests of with varying number of words
Factor score for EF from MMSE, List Learning, Digit Span, Stroop, Clock Drawing, Figure copying, Letter fluency		Factor score for memory from MMSE, List Learning, Digit Span, Stroop, Clock Drawing, Figure copying, Letter fluency

Abbreviations: CANTAB = Cambridge Neuropsychological Test Automated Battery, CERAD = the Consortium to Establish a Registry for Alzheimer's Disease, CVLT = California Verbal Learning Test, HVLT= Hopkins Verbal Learning Test, RVLT = Rey Verbal Learning Test, TICS = Telephone Interview for Cognitive Status, VADAS =the Vascular Dementia Assessment Scale -cognitive subscale, WAIS = Wechsler Adult Intelligence Scale, WAIS-R= Wechsler Adult Intelligence Scale Revised

© 2023 Iso-Markku P et al. *JAMA Network Open.*

1.5 A quality assessment tool for the quality assessment of cohort studies addressing the association of physical activity and dementia or cognition (originally published in British Journal of Sports Medicine: Iso-Markku et al. Br J Sports Med 2022;56:701-709. doi: 10.1136/bjsports-2021-104981)

Note: A study can be given a maximum of one star for each numbered item within the Selection, Comparability and Outcome categories.

Selection
1) Representativeness of the exposed cohort

a) Truly representative (represents well the whole age group or the whole age group of any particular race and is not selected in regards to some disease, socio-economic status or for example only inhabitants of a nursing home, participation rate > 70% (of those alive) and sample size at least 1000 ***(one star)***

b) Truly or somewhat representative (participation rate 50-70%) and sample size > 1000 ***(half a star)***

c) Selected group in regards to some characteristic or participation rate < 50% or sample size < 1000

d) No description of the derivation of the cohort or participation rate lacks

2) Performance quality (adapted from (Br J Sports Med 2017;51:1410-18))

a) Good: PA assessed with a structured questionnaire of the duration, frequency and intensity of PA or the intensity of PA assessed with a structured question. Or PA assessed with an objective measure of PA (eg. accelerometer) **(one star)**

b) Moderate: Participation only in some types of sports assessed but other activities not considered or assessment of intensity lacks. Frequency or duration are assessed. **(half a star)**

c) Low: A "yes" or "no" question used. Frequency and duration not assessed. Or physical activity index on versatility of sports and somewhat physical household chores but not assessing intensity, frequency or duration. Or not described how exercise or physical activity was measured. (no star)

3) Demonstration that outcome of interest was not present at start of study

a) Yes. In a study population whose average age > 55 years, valid measure of cognition is used and demented individuals and individuals with mild cognitive impairment at baseline according to baseline cognition screening have been excluded or population is in midlife (mean age or median < 55 years and maximum age 65 years or +1 SD < 60 years) ***(one star)***

b) No

Comparability
1) Comparability of cohorts on the basis of the design or analysis controlled for confounders

a) The study controls for the following four factors: age, sex (or all cohort members represent the same sex), some vascular risk factor† and education or a measure of general cognitive ability at baseline (education criterion is not needed if all cohort members have the same education level). In addition, the results have been adjusted with baseline cognition in study population whose average age > 55 years **(one star)**

b) The study controls only for three of the factors presented above (age, sex, some vascular risk factor and education or a measure of general intelligence) or/and in study populations whose mean age > 55 years the results have not been adjusted with baseline cognition or the sociodemographic and health behaviors controlled for are not specified further **(half a star)**

c) Cohorts are not comparable on the basis of covariates controlled for (no star)

Outcome
1) Assessment of outcome

a) A validated measure of dementia ***(one star)***

b) Record linkage ***(half a star)***

c) Self report or other

d) No description

2) Was follow-up long enough for outcomes to occur

© 2023 Iso-Markku P et al. *JAMA Network Open.*

a) Yes (***one star)***

b) No

Indicate the median duration of follow-up and a brief rationale for the assessment above: 10 years in dementia studies.

3) Adequacy of follow-up of cohorts

a) Complete follow up- all subject accounted for ***(one star)***

b) Subjects lost to follow up unlikely to introduce bias- number lost less than or equal to 20%* (when follow-up less than 10 years) or 30%* (when follow-up at least 10 years) of those alive or description of those lost suggested no different from those followed. ***(one star)***

c) Follow up rate less than 80%* (when follow-up less than 10 years) or less than 70%* (when follow-up longer than 10 years) and no description of those lost.

d) No statement

* Follow-up rates are calculated taking into account only the cohort members who have been alive at the time of the follow-up
† Vascular risk factor signifies a cardiovascular disease or information on smoking, body mass index, cholesterol levels, diet, blood pressure, diabetes or blood glucose.

Thresholds for converting the Newcastle-Ottawa scales to AHRQ standards (good, moderate, and poor):
Good quality: Selection: 2,5 – 3 stars, Comparability: 1 star, Outcome: 2,5 – 3 stars
Moderate quality: Selection: 2-3 stars, Comparability: 0,5 -1 star, Outcome: 2-3 stars,
Poor quality: studies not reaching Moderate/Good quality

© 2023 Iso-Markku P et al. *JAMA Network Open.*

1.6 A quality assessment tool for the quality assessment of case-control studies addressing the association of physical activity and dementia or cognition (originally published in British Journal of Sports Medicine: Iso-Markku et al. Br J Sports Med 2022;56:701-709. doi: 10.1136/bjsports-2021-104981)

Note: A study can be given a maximum of one star for each numbered item within the Selection, Comparability and Outcome categories.

Baseline
1) Selection of controls:

a) Community controls **(half a star)**

b) Hospital controls

c) No description

2) Definition of controls:

a) No history of disease (endpoint) **(half a star)**

b) No description of source

3) Demonstration that outcome of interest was not present at start of study

a) Yes. In a study population whose average age > 55 years, valid measure of cognition is used and demented individuals and individuals with mild cognitive impairment at baseline according to baseline cognition screening have been excluded or population is in midlife (mean age or median < 55 years and maximum age 65 years or +1 SD < 60 years) **(one star)**

b) No

4) Performance quality (adapted from (Br J Sports Med 2017;51:1410-18))

a) Good: PA assessed with a structured questionnaire of the duration, frequency and intensity of PA or the intensity of PA assessed with a structured question. Or PA assessed with an objective measure of PA (eg. accelerometer). Additionally same method is used for cases and controls and non-response rate is the same for cases and controls. **(one star)**

b) Moderate: Participation only in some types of sports assessed but other activities not considered or assessment of intensity lacks. Frequency or duration are assessed. **(half a star)**

c) Low: A "yes" or "no" question used. Frequency and duration not assessed. Or physical activity index on versatility of sports and somewhat physical household chores but not assessing intensity, frequency or duration. (no star)

Comparability
1) Comparability of cohorts on the basis of the design or analysis controlled for confounders

a) The study controls for the following four factors: age, sex (or all cohort members represent same sex), some vascular risk factor† and education or a measure of general cognitive ability at baseline. In addition, the results have been adjusted with baseline cognition in study population whose average age > 55 years **(one star)**

b) The study controls only for three of the factors presented above (age, sex, some vascular risk factor and education or a measure of general intelligence) or in study populations whose mean age > 55 years the results have not been adjusted with baseline cognition **(half a star)**

c) Cohorts are not comparable on the basis of covariates controlled for (no star)

Outcome
1) Assessment of outcome

a) A validated measure of dementia (if many cognitive tests used, most validated) ***(one star)***

b) Record linkage ***(half a star)***

© 2023 Iso-Markku P et al. *JAMA Network Open.*

c) Self report or other

d) No description

2) Was follow-up long enough for outcomes to occur

a) Yes (***one star)***

b) No

Indicate the median duration of follow-up and a brief rationale for the assessment above: 10 years in dementia studies.

3) Is the case definition adequate?:

a) Yes, with a valid measure of dementia **(half a star)**

b) No, does not fulfil the criteria defined above

4) Representativeness of the cases:

a) Consecutive or obviously representative series of cases **(half a star)**

b) Potential for selection biases or not stated

† Vascular risk factor signifies a cardiovascular disease or information on smoking, body mass index, cholesterol levels, diet, blood pressure, diabetes or blood glucose.

Thresholds for converting the Newcastle-Ottawa scales to AHRQ standards (good, moderate, and poor):
Good quality: Baseline: 2,5 - 3 stars, Comparability: 1 star, Outcome: 2,5 - 3 stars
Moderate quality: Baseline: 2-3 stars, Comparability: 0,5 -1 star, Outcome: 2-3 stars,
Poor quality: studies not reaching Moderate/Good quality

© 2023 Iso-Markku P et al. *JAMA Network Open.*

2. Supplementary analyses

eFigure 1. Quality of the studies

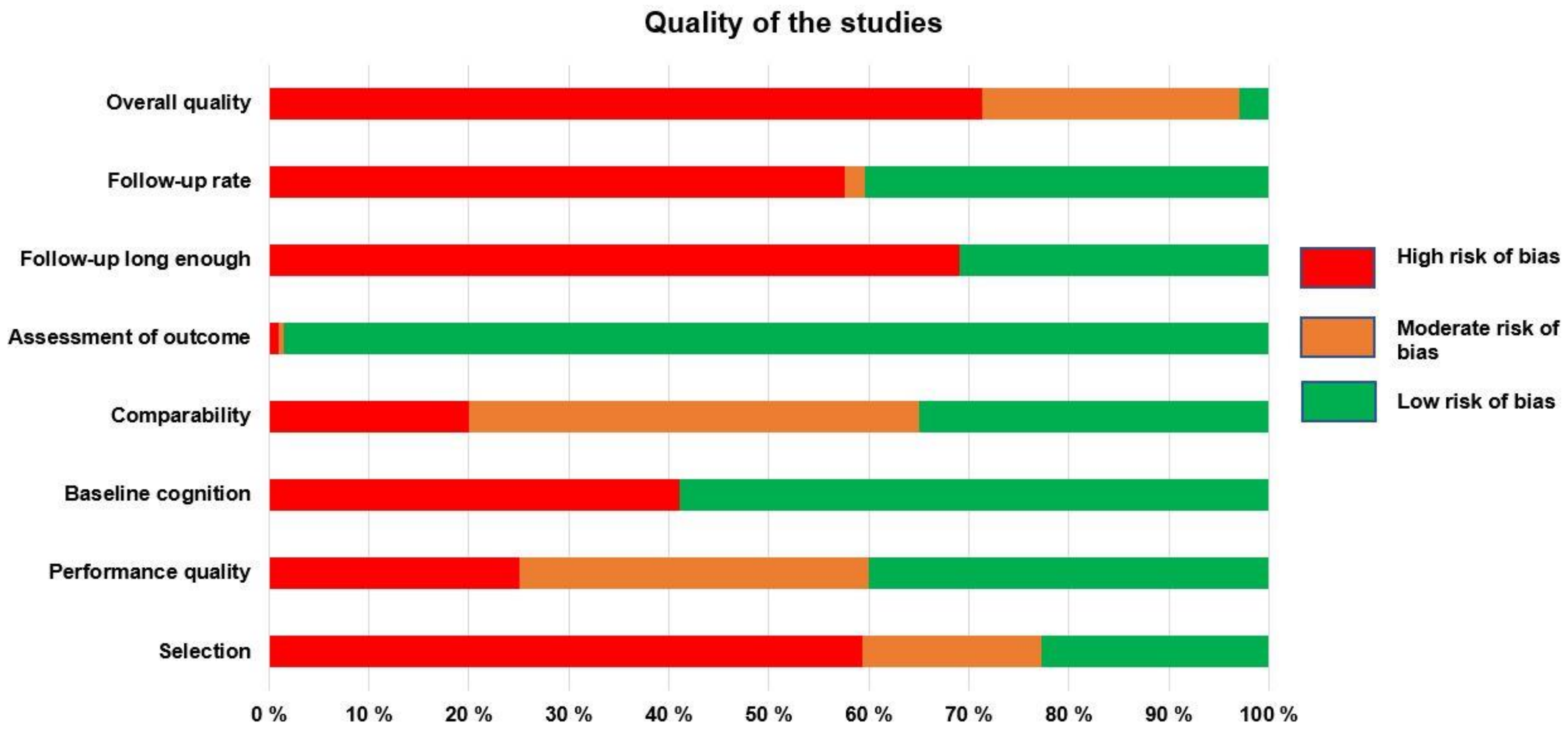

© 2023 Iso-Markku P et al. *JAMA Network Open.*

eTable 5. Physical activity and cognition, binary outcomes, supplementary analyses[a]

[a] Statistically significant results are bolded.

	Pooled RR	95% Confidence interval	I^2	Number of studies combined	Regression coefficient	P value
Highest PA group vs lowest (1 highest vs reference)	**0.90**	**0.87 to 0.93**	**74.2 %**	**45**		
**Moderators**						
Type of PA						
Leisure-time PA and occupational PA not separated	**0.86**	**0.82 to 0.90**	**72.7 %**	**35**		0.13
Only leisure-time PA	**0.92**	**0.88 to 0.97**	**48.3 %**	**10**		
Occupational PA	**0.89**	**0.52 to 1.53**	**90.9 %**	**2**		
Measurement of PA						
Self-report	**0.90**	**0.87 to 0.93**	**65.3 %**	**44**		**<0.001**
Device-measured PA	**0.58**	**0.48 to 0.71**	**8.6 %**	**2**		
Quality of PA measurement [b]					**0.05**	**0.02**
Good (the average rating is one star)	**0.85**	**0.79 to 0.91**	**51.0 %**	**14**		0.11
Moderate (the average rating is 1.5 to 2.5 stars)	**0.88**	**0.84 to 0.93**	**70.7 %**	**18**		
Low (the average rating is no star)	**0.93**	**0.88 to 1.00**	**74.1 %**	**13**		
Sample size					**0.00001**	**0.007**
Number of confounders					-0.002	0.65
Adjustment for education						0.29
No	**0.83**	**0.72 to 0.95**	**61.0 %**	**11**		
Yes	**0.89**	**0.86 to 0.93**	**70.7 %**	**34**		
Adjustment for presence of APOE ε4 allele						0.22
No	**0.89**	**0.86 to 0.93**	**69.9 %**	**38**		
Yes	**0.83**	**0.74 to 0.93**	**29.1 %**	**6**		
Adjustment for chronic disease						0.71
No	**0.87**	**0.79 to 0.94**	**75.0 %**	**17**		
Yes	**0.88**	**0.85 to 0.92**	**67.4 %**	**28**		
Adjustment for other vascular risk factor [c]						0.94
No	**0.88**	**0.83 to 0.94**	**64.6 %**	**20**		
Yes	**0.88**	**0.84 to 0.92**	**72.6 %**	**25**		
Measurement of cognitive impairment						0.15
Dementia screening tool	**0.87**	**0.84 to 0.91**	**72.0 %**	**34**		
At least one neuropsychological test	**0.92**	**0.87 to 0.98**	**51.4 %**	**11**		
Follow-up rate [d]					**-0.007**	**< 0.001**
Follow-up age					0.002	0.44

[b] See “Performance quality” in the quality assessment tool (Supplementary material). One star=1, Half a star=2, No star=3 and if the two reviewers disagreed, then we took the average of the two reviews.

[c] Other vascular risk factor = a cardiovascular disease or information on smoking, BMI, cholesterol levels, diet, blood pressure, diabetes or blood glucose

[d] Drop-out rate has been extracted only by a single reviewer

© 2023 Iso-Markku P et al. *JAMA Network Open.*

eFigure 2. Physical activity and cognitive impairment or decline, forest plot

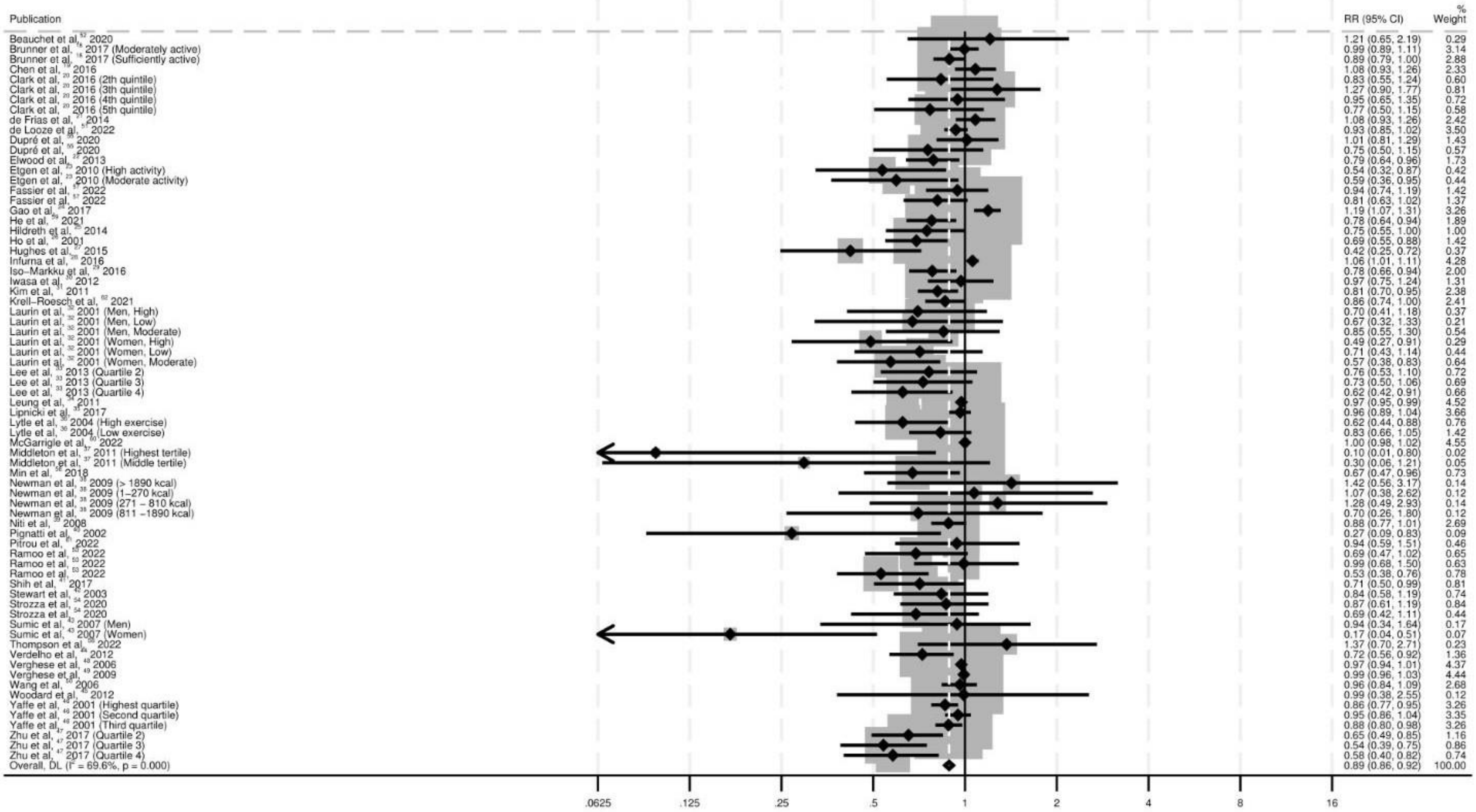

Abbreviations: CI=Confidence interval, I^2=Heterogeneity, p=p-value

© 2023 Iso-Markku P et al. *JAMA Network Open.*

eFigure 3. Physical activity and cognition, binary outcome, contour-enhanced funnel plot

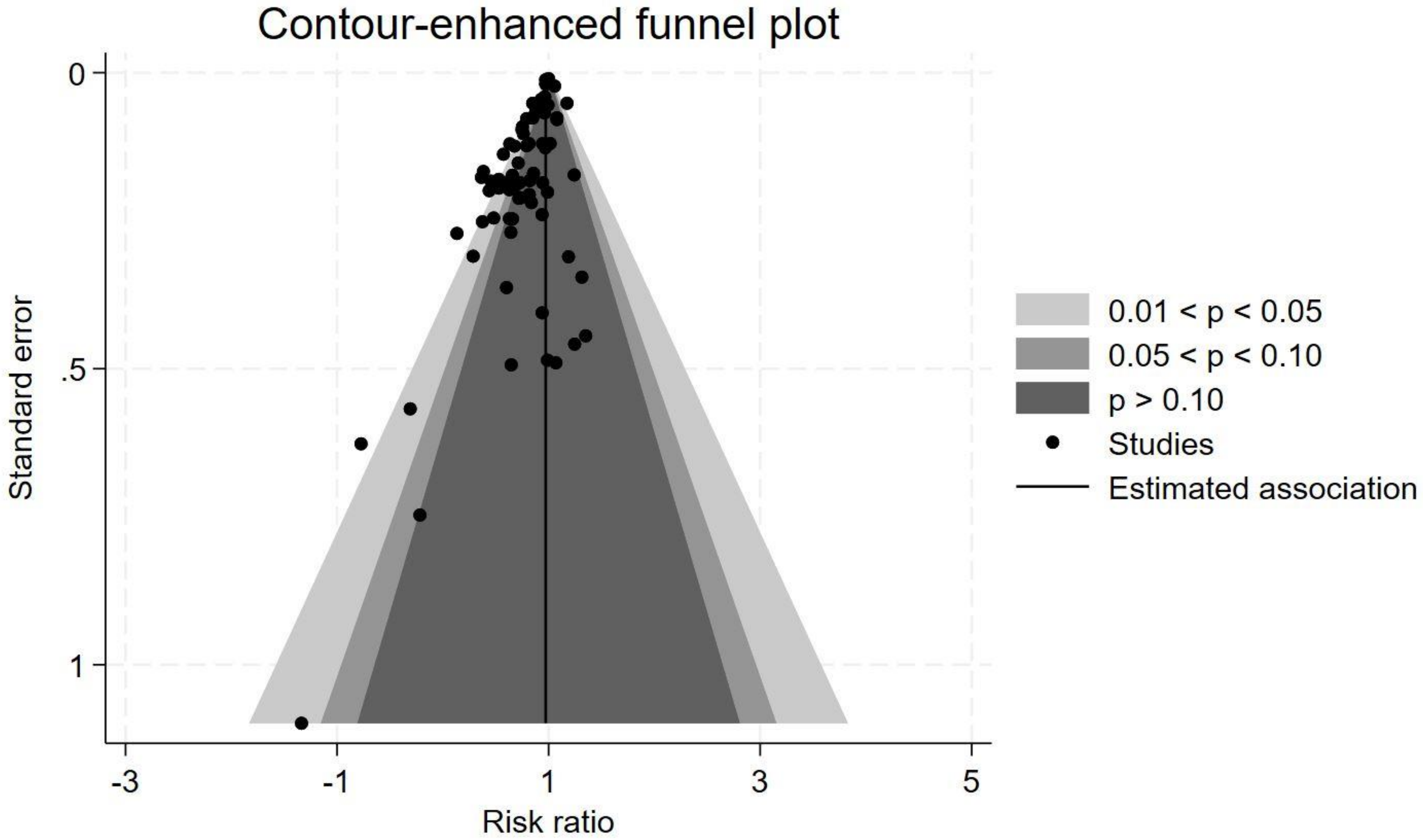

Abbreviations: p=p-value

© 2023 Iso-Markku P et al. *JAMA Network Open.*

eFigure 4. Physical activity and cognition, binary outcomes by sample size

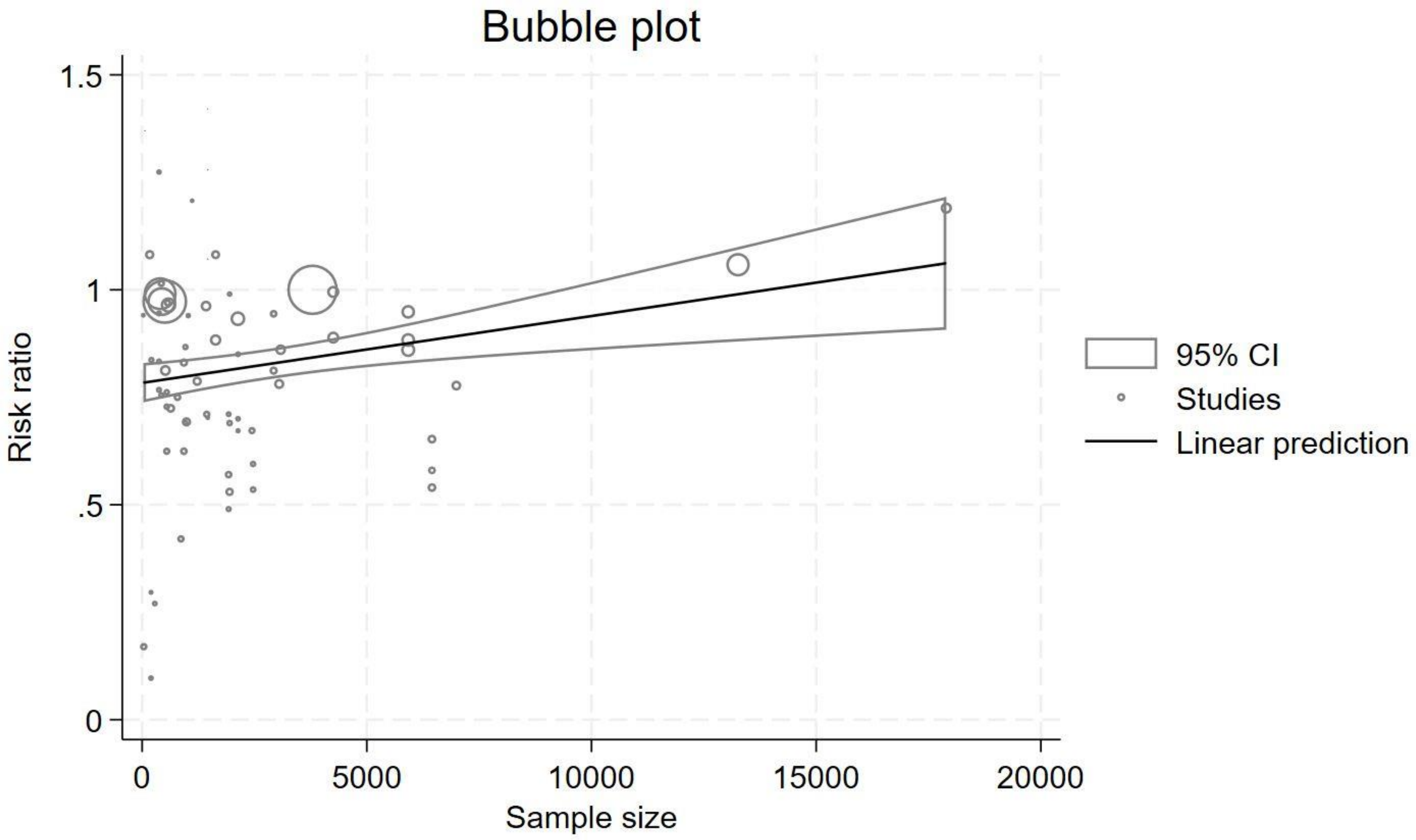

Abbreviations: CI=Confidence interval

© 2023 Iso-Markku P et al. *JAMA Network Open.*

eFigure 5. Physical activity and cognition, binary outcomes by length of the follow-up

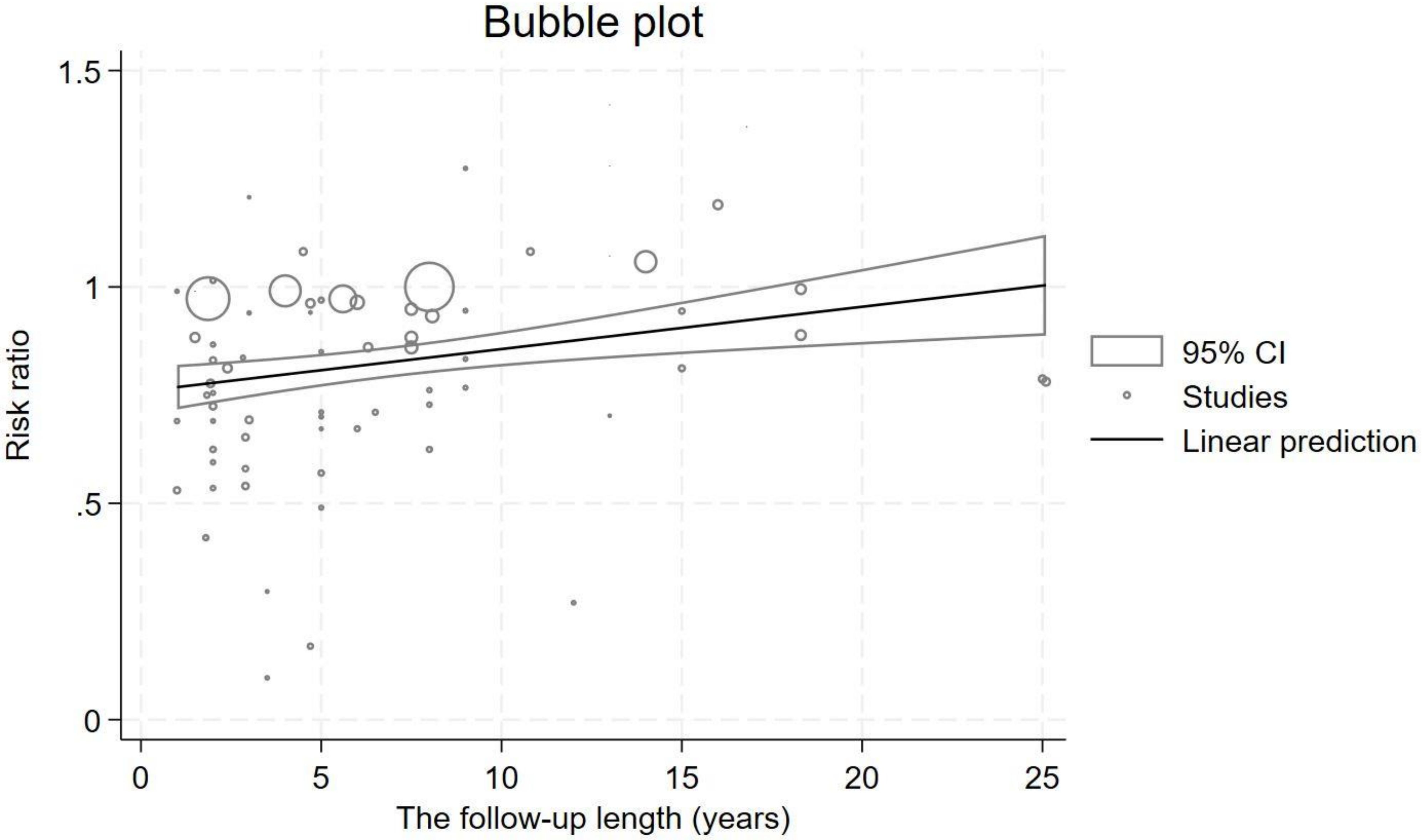

Abbreviations: CI=Confidence interval

© 2023 Iso-Markku P et al. *JAMA Network Open.*

eFigure 6. Physical activity and cognition, binary outcomes by follow-up rate (studies not reporting follow-up rate are excluded)

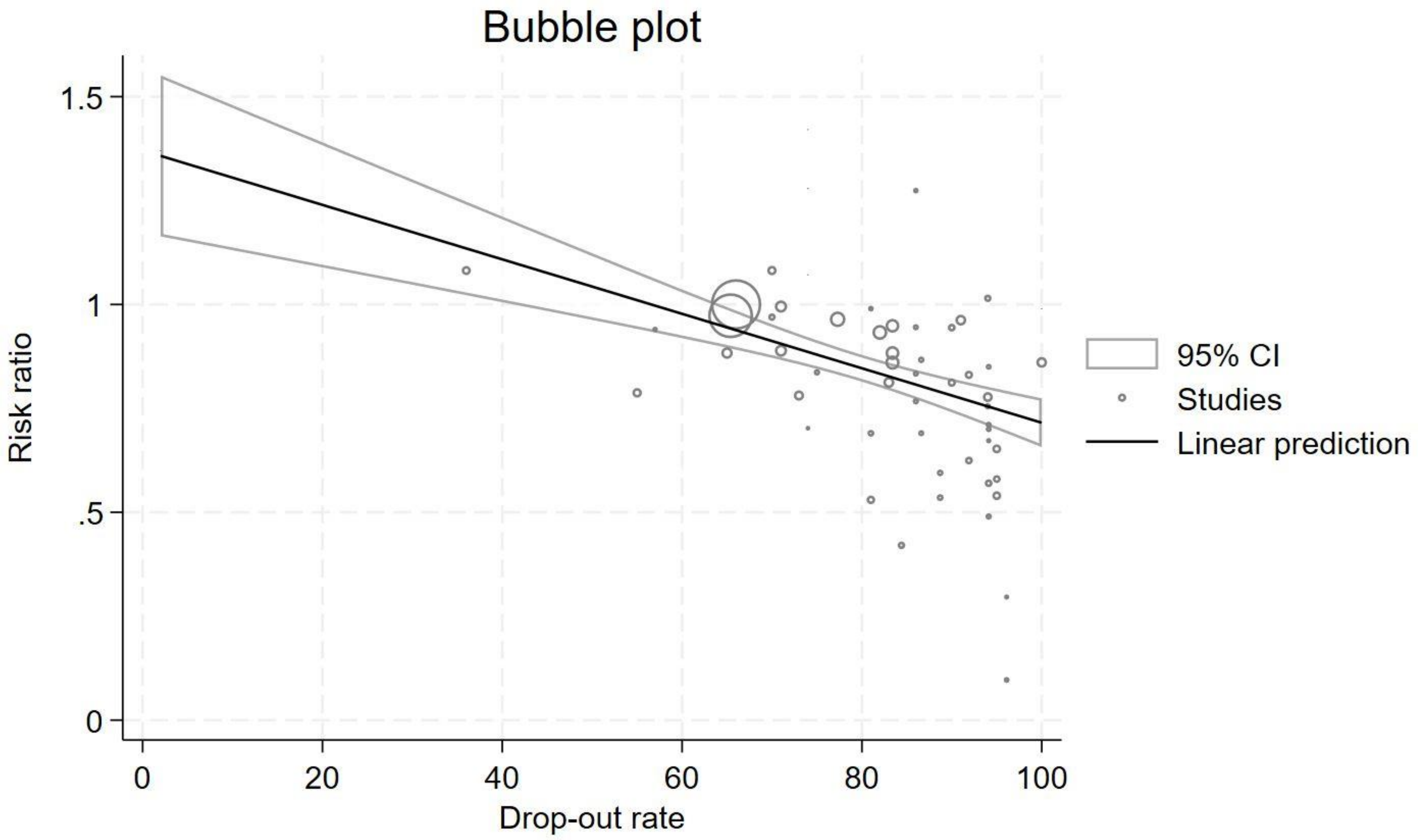

Abbreviations: CI=Confidence interval

© 2023 Iso-Markku P et al. *JAMA Network Open.*

eTable 6. Physical activity and follow-up cognition: supplementary analyses [a]

[a] Statistically significant results are bolded.

	Pooled standardized regression coefficient	95% Confidence interval	I^2	Number of studies combined	Regression coefficient	P value
**Moderators**						
Type of PA						0.66
Occupational PA	-	-	-	-		
Only leisure-time PA	**0.029**	**0.014 to 0.044**	**40.1 %**	**5**		
Leisure-time PA and occupational PA not separated	0.018	-0.028 to 0.064	83.2 %	9		
Measurement of PA						0.25
Self-report	**0.032**	**0.012 to 0.052**	**70.6 %**	**13**		
Device-measured PA	-0.083	-0.278 to 0.111	21.1 %	2		
Quality of PA measurement [b]					0.01	0.55
Sample size					1.1e-7	0.98
Number of confounders					-0.001	0.30
Adjustment for education						0.70
No	0.053	-0.091 to 0.197	83.0 %	2		
Yes	**0.025**	**0.002 to 0.047**	**76.7 %**	**12**		
Adjustment for presence of APOE ε4 allele						0.49
No	**0.029**	**0.008 to 0.051**	**70.7 %**	**12**		
Yes	-0.03	-0.199 to 0.138	94.3 %	2		
Adjustment for chronic disease						0.68
No	0.017	-0.031 to 0.065	83.6 %	5		
Yes	**0.028**	**0.002 to 0.054**	**72.7 %**	**9**		
Adjustment for other vascular risk factor [c]						0.62
No	0.009	-0.074 to 0.091	85.5 %	4		
Yes	**0.030**	**0.009 to 0.052**	**72.7 %**	**11**		
Measurement of cognition						0.06
Dementia screening tool	**0.043**	**0.013 to 0.072**	**76.8 %**	**8**		
Follow-up rate					-0.0006	0.21
Follow-up age					0.0000	0.95
Less than 60 years	**0.017**	**-0.015 to 0.049**	**67.0 %**			
At least 60 years	0.032	-0.000 to 0.064	79.9 %			

[b] See "Performance quality" in the quality assessment tool (Supplementary material). One star=1, Half a star=2, No star=3 and if the two reviewers disagreed, then we took the average of the two reviews.

[c] Other vascular risk factor = a cardiovascular disease or information on smoking, BMI, cholesterol levels, diet, blood pressure, diabetes or blood glucose.

© 2023 Iso-Markku P et al. *JAMA Network Open.*

eFigure 7. PA and follow-up cognition, contour-enhanced funnel plot

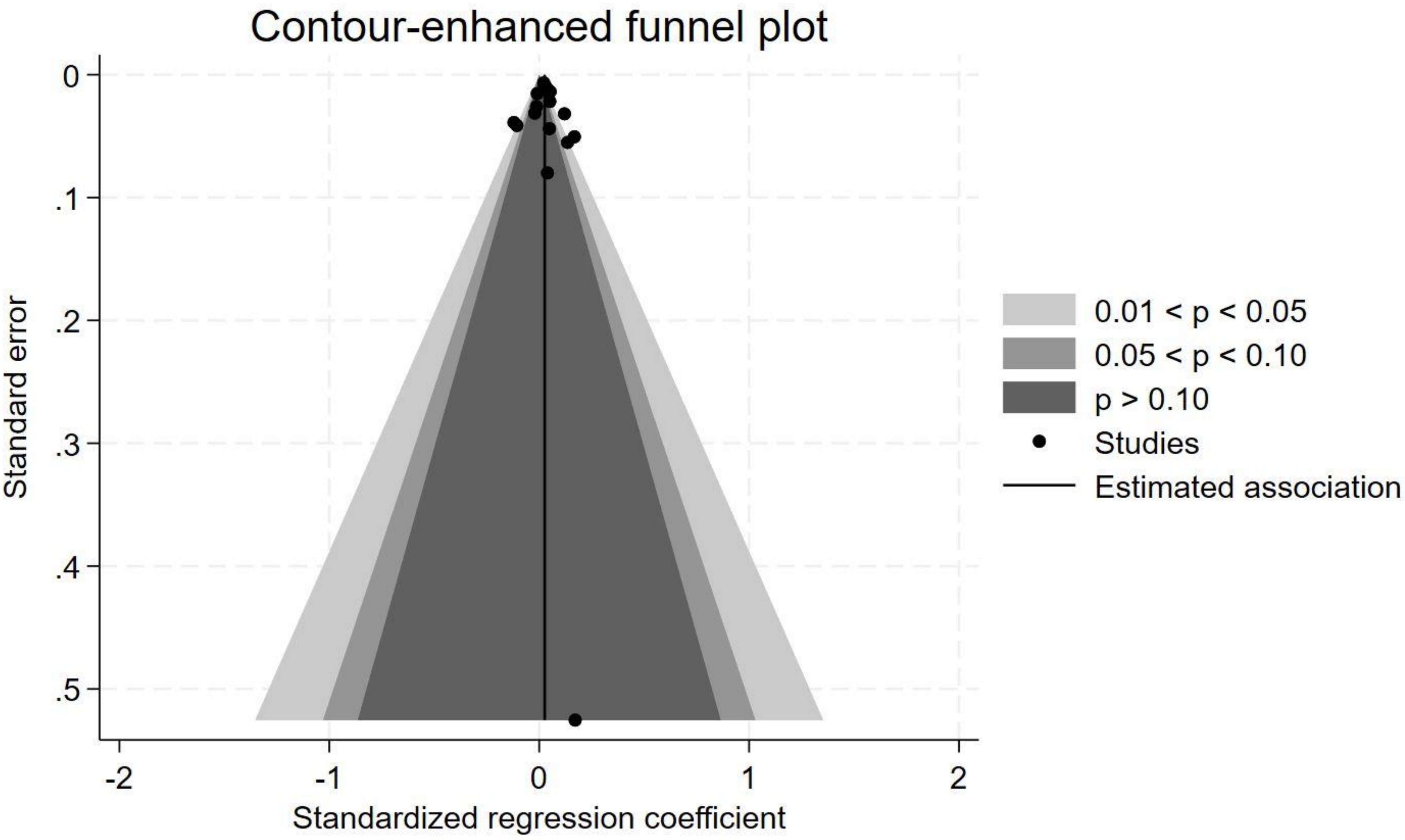

Abbreviations: p=p-value

© 2023 Iso-Markku P et al. *JAMA Network Open.*

eTable 7. PA and change in cognition: supplementary analyses [a]

	Pooled standardized regression coefficient	95% Confidence interval	I^2	Number of studies combined	Regression coefficient	P value
**Moderators**						
Type of PA						0.07
Occupational PA	-	-	-	-		
Only leisure-time PA	-0.004	-0.033 to 0.025	83.6 %	8		
Leisure-time PA and occupational PA not separated	**0.026**	**0.012 to 0.040**	**35.0 %**	**17**		
Measurement of PA						0.44
Self-report	**0.016**	**0.002 to 0.029**	**68.5 %**	**24**		
Device-measured PA	0.051	-0.037 to 0.139	-	1		
Quality of PA measurement [b]					0.0008	0.94
Sample size					-2.4e-7	0.87
Number of confounders					0.002	0.10
Adjustment for education						0.70
No	0.054	-0.136 to 0.244	85.0 %	3		
Yes	**0.017**	**0.005 to 0.030**	**63.2 %**	**22**		
Adjustment for presence of APOE ε4 allele						0.42
No	0.014	-0.001 to 0.029	69.4 %	21		
Yes	0.036	-0.014 to 0.086	62.3 %	4		
Adjustment for chronic disease						0.53
No	0.006	-0.030 to 0.042	56.1 %	10		
Yes	**0.019**	**0.003 to 0.034**	**73.6 %**	**15**		
Adjustment for other vascular risk factor [c]						0.82
No	0.016	-0.003 to 0.035	58.1 %	12		
Yes	0.020	-0.005 to 0.044	74.2 %	13		
Measurement of cognition						0.35
Dementia screening tool	0.004	-0.031 to 0.040	71.1 %	12		
At least one neuropsychological test	**0.022**	**0.009 to 0.036**	**63.1 %**	**13**		
Follow-up rate					0.004	0.61
Follow-up age (in years)					-0.001	0.34

[a] Statistically significant results are bolded.

[b] See "Performance quality" in the quality assessment tool (Supplementary material). One star=1, Half a star=2, No star=3 and if the two reviewers disagreed, then we took the average of the two reviews.

[c] Other vascular risk factor = a cardiovascular disease or information on smoking, BMI, cholesterol levels, diet, blood pressure, diabetes or blood glucose

© 2023 Iso-Markku P et al. *JAMA Network Open.*

eFigure 8. PA and change in cognition, contour-enhanced funnel plot

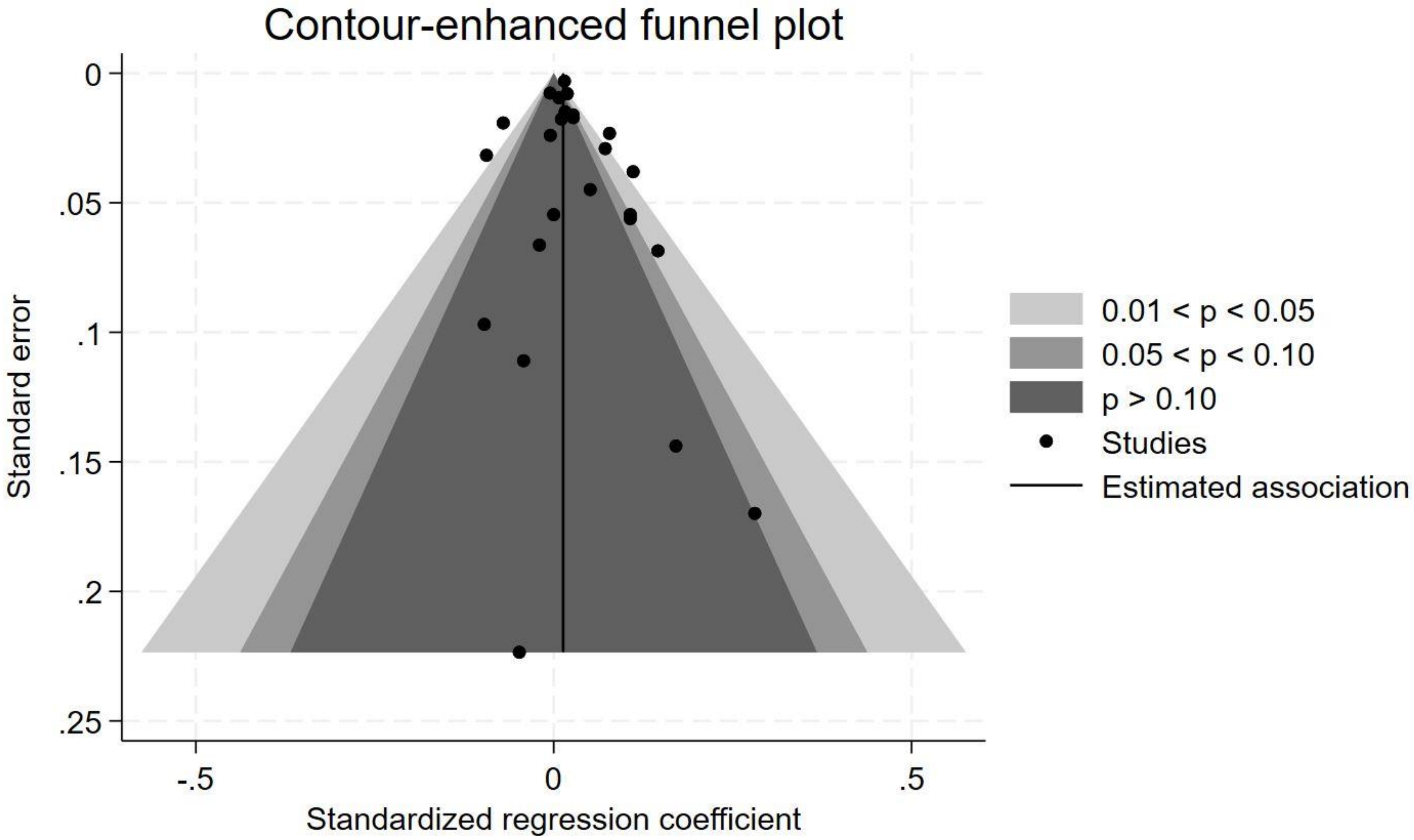

Abbreviations: p=p-value

© 2023 Iso-Markku P et al. *JAMA Network Open.*

eTable 8. PA and specific cognitive domains in studies adjusting for preceding level of cognition (global or specific)[a]

	Risk ratio	95 % Confidence intervals	I^2	Number of studies combined	Number of persons	Weighted average follow-up length	Weighted average baseline age
**Binary outcomes**							
Executive function	1.11	0.94 to 1.32	95.7 %	3	7 399	9.1	62.4
Episodic memory	0.94	0.88 to 1.00	0 %	4	10 419	10.0	65.6
Processing speed [b]	0.98	0.79 to 1.22	32.0 %	1	376	9.0	73.8
Verbal fluency [c]	0.92	0.82 to 1.03	58.9 %	2	16 082	12.0	49.4
Working memory	-	-	-	-	-	-	-
Verbal ability	-	-	-	-	-	-	-
Visuospatial ability	**0.99**	**0.97 to 1.00**	**-**	**1**	67	2.5	71.1
**Follow-up cognition**	**Standardized regression coefficient**						
Executive function	**0.052**	**0.013 to 0.092**	**56.7 %**	**6**	18 109	15.2	47.8
Episodic memory	**0.025**	**0.016 to 0.035**	**54.6 %**	**12**	175 298	6.0	63.8
Processing speed	0.030	-0.013 to 0.072	0%	7	7 583	8.7	59.3
Verbal fluency	**0.053**	**0.025 to 0.080**	**38.4 %**	**5**	108 736	2.3	67.3
Working memory [b]	0.001	-0.013 to 0.072	0 %	4	4 681	8.6	15.7
Verbal ability [b]	**0.036**	**0.011 to 0.060**	**-**	**1**	5 197	8.0	34.0
Visuospatial ability [b]	0.022	-0.043 to 0.087	0 %	2	1 496	16.3	26.0
**Change in cognition**	**Standardized regression coefficient**						
Executive function	0.013	-0.001 to 0.027	40.3 %	12	23 520	6.3	63.8
Episodic memory	**0.024**	**0.012 to 0.035**	**51.3 %**	**20**	53 645	5.5	68.3
Processing speed	0.041	-0.053 to 0.134	39.4 %	6	5 607	6.1	71.3
Verbal fluency	**0.021**	**0.004 to 0.039**	**67.0 %**	**11**	22 190	8.6	64.1
Working memory	-0.003	-0.066 to 0.060	0 %	2	3 198	2.1	76.7
Verbal ability	-0.000	-0.000 to 0.000	0 %	2	2 484	9.4	70.7
Visuospatial ability	0.023	-0.025 to 0.070	76.6 %	3	3 814	8.9	69.0

[a] Statistically significant results are bolded.
[b] The result of only one study, not a meta-analysis.
[c] The results for studies adjusting for preceding level of cognition or not were significantly different from each other for binary outcomes and change in cognition. Binary outcomes not adjusting for preceding level of cognition: pooled RR 0.75 (95% CI: 0.63 to 0.88, I^2=0%, number of studies: 1) and studies adjusting for preceding level of cognition: pooled RR 0.98 (95 % CI: 0.90 to 1.07, I^2=12.3 %, number of studies: 1). Studies addressing PA and change in cognition not adjusting for preceding level of cognition: pooled standardized regression coefficient 0.001 (95% CI: -0.002 to 0.005, I^2=0%, number of studies: 7) and adjusting for preceding level of cognition: pooled standardized regression coefficient 0.033 (95% CI: 0.021 to 0.046, I^2=0%, number of studies:4).

© 2023 Iso-Markku P et al. *JAMA Network Open.*

eReferences

1. Iso-Markku P, Kujala UM, Knittle K, et al. Physical activity as a protective factor for dementia and Alzheimer's disease: systematic review, meta-analysis and quality assessment of cohort and case-control studies. *Br J Sports Med* 2022;56:701-9. doi:10.1136/bjsports-2021-104981
2. Salthouse TA. Correlates of cognitive change. *J Exp Psychol Gen* 2014;143:1026-48. doi:10.1037/a0034847
3. Zhang J, Yu KF. What's the relative risk? A method of correcting the odds ratio in cohort studies of common outcomes. *JAMA* 1998;280:1690-1. doi:10.1001/jama.280.19.1690
4. VanderWeele TJ. On a Square-Root Transformation of the Odds Ratio for a Common Outcome. *Epidemiology* 2017;28:e58-e60. doi:10.1097/ede.0000000000000733
5. Shor E, Roelfs D, Vang ZM. The "Hispanic mortality paradox" revisited: Meta-analysis and meta-regression of life-course differentials in Latin American and Caribbean immigrants' mortality. *Soc Sci Med* 2017;186:20-33. doi:10.1016/j.socscimed.2017.05.049
6. Altman DG, Bland JM. How to obtain the confidence interval from a P value. *BMJ* 2011;343:d2090. doi:10.1136/bmj.d2090
7. McGarrigle CA, Ward M, Kenny RA. Negative aging perceptions and cognitive and functional decline: Are you as old as you feel? *J Am Geriatr Soc* 2022;70:777-88. doi:10.1111/jgs.17561
8. De Looze C, Williamson W, Demnitz N, et al. Physical Function, An Adjunct to Brain Health Score for Phenotyping Cognitive Function Trajectories in Older Age: Findings From The Irish Longitudinal Study on Ageing (TILDA). *J Gerontol A Biol Sci Med Sci.* 2022;77:1593-602. doi:10.1093/gerona/glac024
9. Fassier P, Kang JH, Lee IM, et al. Vigorous Physical Activity and Cognitive Trajectory Later in Life: Prospective Association and Interaction by Apolipoprotein E e4 in the Nurses' Health Study. *J Gerontol A Biol Sci Med Sci* 2022;77:817-25. doi:10.1093/gerona/glab169
10. Min JW. A longitudinal study of cognitive trajectories and its factors for Koreans aged 60 and over: A latent growth mixture model. *Int J Geriatr Psychiatry* 2018;33:755-62. doi:10.1002/gps.4855
11. Chen TY, Chang HY. Developmental Patterns of Cognitive Function and Associated Factors among the Elderly in Taiwan. *Sci Rep* 2016;6:33486. doi:10.1038/srep33486
12. Stenling A, Sörman DE, Lindwall M, et al. Bidirectional Within- and Between-Person Relations Between Physical Activity and Cognitive Function. *J Gerontol B Psychol Sci Soc Sci.* 2022;7:704-9. doi:10.1093/geronb/gbab234
13. Feingold A. A Regression Framework for Effect Size Assessments in Longitudinal Modeling of Group Differences. *Rev Gen Psychol* 2013;17:111-21. doi:10.1037/a0030048
14. Nieminen P. Application of Standardized Regression Coefficient in Meta-analysis. *BioMedInformatics* 2022;2:434-58. doi:https://doi.org/10.3390/biomedinformatics2030028
15. Graham PR, LM; Luszcz, MA. Joint modelling of survival and cognitive decline in the Australian Longitudinal Study of Ageing. *J R Stat Soc C Appl Stat.* 2011;60.
16. Brody DJ, Kramarow EA, Taylor CA, et al. Cognitive Performance in Adults Aged 60 and Over: National Health and Nutrition Examination Survey, 2011-2014. *Natl Health Stat Report* 2019:1-23.
17. Wilson D. Formulas Used by the "Practical Meta-analysis Effect Size Calculator". 2017.
18. Blond K, Brinkløv CF, Ried-Larsen M, et al. Association of high amounts of physical activity with mortality risk: a systematic review and meta-analysis. *Br J Sports Med* 2020;54:1195-201. doi:10.1136/bjsports-2018-100393
19. Walpole SC, Prieto-Merino D, Edwards P, et al. The weight of nations: an estimation of adult human biomass. *BMC Public Health* 2012;12:439. doi:10.1186/1471-2458-12-439
20. Bull FC, Al-Ansari SS, Biddle S, et al. World Health Organization 2020 guidelines on physical activity and sedentary behaviour. *Br J Sports Med* 2020;54:1451-62. doi:10.1136/bjsports-2020-102955
21. Nilsson LG, Bäckman L, de Frias CM, Molander B, Nyberg L. Betula: A prospective cohort study on memory, health and aging. *Aging Neuropsychol Cogn.* 2004;11:134-48.

© 2023 Iso-Markku P et al. *JAMA Network Open.*
