# Supplementary material for: Physical Activity and Cognitive Decline Among Older Adults: A Systematic Review and Meta-Analysis
Source: JAMA Netw Open. 2024 Feb 1;7(2):e2354285. doi: 10.1001/jamanetworkopen.2023.54285 (PMC10835510; doi:10.1001/jamanetworkopen.2023.54285)
Supplement: Supplement 3. — Data Sharing Statement [file jamanetwopen-e2354285-s003.pdf]

Data Sharing Statement

Iso-Markku. Physical Activity and Cognitive Decline Among Older Adults. *JAMA Netw Open*. Published February 01, 2024. doi:10.1001/jamanetworkopen.2023.54285

Data
Data available: Yes
Data types: Other (please specify)
Additional Information: Data extracted from other articles.
How to access data: Data extracted from other articles.
When available: With publication

Supporting Documents
Document types: None

Additional Information
Who can access the data: All readers of the article.
Types of analyses: All readers of the article can access the data extracted and collected from from other articles for the purposes of this meta-analysis.
Mechanisms of data availability: The data extracted is in the Supplement 2.
